# Postoperative circulating tumor DNA as markers of recurrence risk in stages II to III colorectal cancer

**DOI:** 10.1186/s13045-021-01089-z

**Published:** 2021-05-17

**Authors:** Gong Chen, Junjie Peng, Qian Xiao, Hao-Xiang Wu, Xiaojun Wu, Fulong Wang, Liren Li, Peirong Ding, Qi Zhao, Yaqi Li, Da Wang, Yang Shao, Hua Bao, Zhizhong Pan, Ke-Feng Ding, Sanjun Cai, Feng Wang, Rui-Hua Xu

**Affiliations:** 1 Department of Colorectal Surgery, State Key Laboratory of Oncology in South China, Collaborative Innovation Center for Cancer Medicine, Sun Yat-Sen University Cancer Center, Guangzhou, 510060 China; 2 Department of Medical Oncology, State Key Laboratory of Oncology in South China, Collaborative Innovation Center for Cancer Medicine, Sun Yat-Sen University Cancer Center, 651 Dongfeng Road East, Guangzhou, 510060 China; 3 Department of Colorectal Surgery, Fudan University Shanghai Cancer Center, Shanghai, 200032 China; 4 Department of Colorectal Surgery and Oncology, Key Laboratory of Cancer Prevention and Intervention, Ministry of Education, The Second Affiliated Hospital, Zhejiang University School of Medicine, Hangzhou, 310009 China; 5 Department of Oncology, Shanghai Medical College, Fudan University, Shanghai, 200032 China; 6 Cancer Center Zhejiang University, Hangzhou, 310009 China; 7 Department of Experimental Research, State Key Laboratory of Oncology in South China, Collaborative Innovation Center for Cancer Medicine, Sun Yat-Sen University Cancer Center, Guangzhou, 510060 China; 8 Nanjing Geneseeq Technology Inc., Nanjing, 210032 China; 9 School of Public Health, Nanjing Medical University, Nanjing, 210029 China; 10 Research Unit of Precision Diagnosis and Treatment for Gastrointestinal Cancer, Chinese Academy of Medical Sciences, Guangzhou, 510060 China

**Keywords:** Stage II/III colorectal cancer, Minimal residual disease, Circulating tumor DNA, Recurrence risk, Adjuvant chemotherapy

## Abstract

**Background:**

Precise methods for postoperative risk stratification to guide the administration of adjuvant chemotherapy (ACT) in localized colorectal cancer (CRC) are still lacking. Here, we conducted a prospective, observational, and multicenter study to investigate the utility of circulating tumor DNA (ctDNA) in predicting the recurrence risk.

**Methods:**

From September 2017 to March 2020, 276 patients with stage II/III CRC were prospectively recruited in this study and 240 evaluable patients were retained for analysis, of which 1290 serial plasma samples were collected. Somatic variants in both the primary tumor and plasma were detected via a targeted sequencing panel of 425 cancer-related genes. Patients were treated and followed up per standard of care.

**Results:**

Preoperatively, ctDNA was detectable in 154 of 240 patients (64.2%). At day 3–7 postoperation, ctDNA positivity was associated with remarkably high recurrence risk (hazard ratio [HR], 10.98; 95%CI, 5.31–22.72; *P* < 0.001). ctDNA clearance and recurrence-free status was achieved in 5 out of 17 ctDNA-positive patients who were subjected to ACT. Likewise, at the first sampling point after ACT, ctDNA-positive patients were 12 times more likely to experience recurrence (HR, 12.76; 95%CI, 5.39–30.19; *P* < 0.001). During surveillance after definitive therapy, ctDNA positivity was also associated with extremely high recurrence risk (HR, 32.02; 95%CI, 10.79–95.08; *P* < 0.001). In all multivariate analyses, ctDNA positivity remained the most significant and independent predictor of recurrence-free survival after adjusting for known clinicopathological risk factors. Serial ctDNA analyses identified recurrence with an overall accuracy of 92.0% and could detect disease recurrence ahead of radiological imaging with a mean lead time of 5.01 months.

**Conclusions:**

Postoperative serial ctDNA detection predicted high relapse risk and identified disease recurrence ahead of radiological imaging in patients with stage II/III CRC. ctDNA may be used to guide the decision-making in postsurgical management.

**Supplementary Information:**

The online version contains supplementary material available at 10.1186/s13045-021-01089-z.

## Background

Colorectal cancer (CRC) is the third most common cancer around the world with more than 1.9 million new cases diagnosed annually [1]. CRC is also the second leading cause of cancer-related deaths [1] with a 5-year mortality rate of about 40% [2], which is also a great health burden in China [3, 4]. Through the implementation of screening by serum carcinoembryonic antigen (CEA) and colonoscopy, increasing CRC patients could nowadays be diagnosed at an earlier stage before the formation of metastatic lesions [5, 6], for whom surgical resection is the optimal treatment modality. However, a substantial proportion of patients still experience disease recurrence after the curative resection.

The existence of minimal residual disease (MRD), which is clinically occult and radiologically invisible at the time of surgery, has been considered the major source of disease recurrence [7]. Therefore, a standard of care of 3- to 6-month adjuvant chemotherapy (ACT) has been widely adopted to eradicate the MRD in patients with clinicopathological high-risk factors, including stage III, poor differentiation, lymphovascular invasion, nerve invasion, and so on [8, 9]. However, as not all patients with these high-risk factors have MRD after surgery, a considerable number of patients have to suffer from the adverse effects of ACT without clinical benefit. In contrast, for patients without high-risk factors, MRD may still exist and thus some of them could potentially benefit from ACT. In addition, 20–30% patients who received ACT still experience disease recurrence [10, 11], but there is no available tool to evaluate the efficacy of ACT and guide the post-ACT management.

The direct and real-time measurement of MRD is an ideal solution to facilitate the decision-making in postsurgical management. Detection of circulating tumor DNA (ctDNA) holds great promise for this issue. ctDNA, a fraction of a patient’s total circulating free DNA (cfDNA), is shed into the bloodstream by the breakdown of tumor cells and thus could reflect the disease burden [12]. Therefore, we could theoretically catch the radiologically invisible MRD by detecting the ctDNA it releases [13]. Several recent studies used multiplex PCR-based next-generation sequencing (NGS) of highly selected somatic variants for ctDNA detection in a relatively small number (~ 100) of resectable CRC patients and revealed the association between postsurgical ctDNA detection and disease recurrence [14–17]. However, the technical complexity and long turnaround time of this customized approach may impede its routine clinical application in the setting of the time-sensitive decision-making for postoperative ACT administration.

Herein, we report the results of a prospective, observational and multicenter study using a 425-gene NGS panel-based approach to measure ctDNA and evaluating its association with disease recurrence in 240 patients with stage II/III CRC. Our work indicates that the detection of ctDNA could reflect the existence of MRD, and ctDNA evaluation as early as 3–7 days postoperatively may facilitate risk stratification and decision-making in postsurgical management.

## Methods

### Study design and participants

This study recruited patients with stage II/III CRC from September 2017 to March 2020 at Fudan University Shanghai Cancer Center, the Second Affiliated Hospital of Zhejiang University School of Medicine and Sun Yat-sen University Cancer Center. This project was approved by the ethics committees at each hospital and was performed in accordance with the Declaration of Helsinki. All participants provided written informed consent. Tumor tissue was collected at surgery, serial blood samples were collected preoperatively within 7 days, postoperatively at day 3–7 before discharge, 6 months after surgery, and then every 3 months until month 24 unless the patient passed away or withdrew informed consent. All patients were treated and followed up according to the Chinese Society of Clinical Oncology guideline [18]. The use of ACT after surgery was at the discretion of the clinicians and patients, both were blinded to the ctDNA results. Clinical follow-up included clinical review and serum CEA test for every 3 months, and annual CT scan. Clinicopathological data, as well as postoperative follow-up and surveillance information, was also collected (Additional file 1: Table S1).

### Circulating tumor DNA analysis

The peripheral blood leukocytes, primary tumors and plasma samples were all targeted-sequenced by the Geneseeq Prime™ 425-gene panel (Additional file 2: Table S2), yielding a mean sequencing depth of 276 ×, 1277 × and 4693 ×, respectively. All qualified variants identified in the primary tumor of each patient were regarded as patient-specific somatic variants for further ctDNA tracking. A plasma sample was declared as ctDNA-positive if the number of true variants detected in the plasma was more than 5% of the number of total tracking variants in each patient. For details, see Additional file 3: Methods.

### Statistical analysis

The primary outcome was recurrence-free survival (RFS) assessed by standard radiological criteria. RFS was calculated from the date of surgery to the date of verified radiological recurrence or death as a result of CRC for patients who relapsed and was censored at last follow-up or non-CRC-related death for patients who were not documented with recurrence. RFS analysis, univariate, and multivariate analysis were performed using the Kaplan–Meier estimator and Cox proportional hazard regression analysis. Statistical analyses were performed using the R software v3.6.2 (https://www.r-project.org/), and a two-sided *P* value < 0.05 was considered significant.

## Results

### Clinicopathological characteristics of the study cohort

**Fig. 1:**
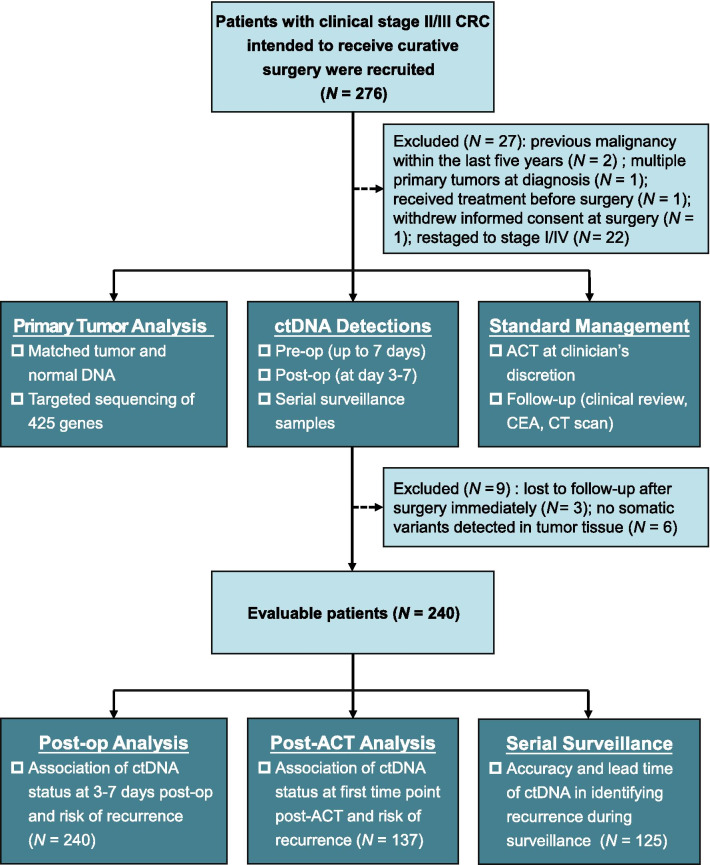
Flowchart depicting the patient enrollment, sample collections and evaluable population. ACT, adjuvant chemotherapy; CEA, carcinoembryonic antigen; CRC, colorectal cancer; CT, computed tomography; ctDNA, circulating tumor DNA; pre-op, pre-operation; post-op, postoperation

A total of 276 patients diagnosed with clinical stage II/III CRC were recruited at the study entry, and 240 evaluable patients were retained for analysis (Fig. 1). The clinicopathological characteristics of 240 evaluable patients were summarized in Additional file 4: Table S3. The median age was 60 years (range 19–84) and 43.3% of the patients were female. About one-third of the patients (87/240, 36.3%) had right-sided CRC (from cecum to splenic flexure) and the other two-thirds (153/240, 63.7%) had left-sided CRC (from splenic flexure to rectum). 46.7% (112/240) of the patients were classified as pathological stage II and the rest (128/240, 53.3%) were stage III. Almost all of the stage III patients (121/128, 94.5%) received at least one dose of ACT while 47.3% (53/112) of the stage II patients did. During a median follow-up time of 27.4 months (95% CI 26.2–28.5), 32 patients were recorded to experience radiological recurrence, including 10 stage II patients and 22 stage III patients.

In the primary tumors, the number of identified somatic variants ranged from 1 to 327 (median, 6). The detailed mutational landscape is presented in Additional file 5: Figure S1 together with corresponding clinicopathological features (for detailed list of somatic mutations, see Additional file 6: Dataset S1). For these 240 patients, a total of 1290 plasma samples were collected and analyzed, with a median of five plasma samples for each patient (for detailed ctDNA profiling results, see Additional file 7: Dataset S2).

CEA was used as the standard-of-care blood test for screening and monitoring of CRC. However, CEA was elevated in only 91 of 231 (39.4%) preoperative plasma samples (Additional file 8: Figure S2, detailed CEA results are presented in Additional file 9: Dataset S3). By contrast, in the 240 preoperative plasma samples, ctDNA was detected in 154 of 240 samples (64.2%), with a sensitivity of 65.2% in stage II CRC and 63.3% in stage III CRC (Additional file 8: Figure S2), which was much higher than that of CEA.

No significant RFS difference was observed between patients with elevated CEA preoperatively versus those without (HR 1.53; 95% CI 0.74–3.18; *P* = 0.250; Additional file 10: Figure S3); while preoperative ctDNA-positive patients had reduced RFS compared with that of preoperative ctDNA-negative patients (HR 5.66; 95% CI 1.72–18.57; *P* = 0.004; Additional file 10: Figure S3). The clinical features between the preoperative ctDNA-positive and ctDNA-negative subsets are similar (Additional file 11: Table S4), indicating that the RFS difference maybe attributed to that patients with greater metastatic potential may have higher ctDNA concentration and thus were enriched in the ctDNA-positive subset. To avoid potential bias, we adjusted for the preoperative ctDNA status in the following multivariate analysis.

### Postoperative ctDNA status in 1 week and its association with recurrence risk

**Fig. 2:**
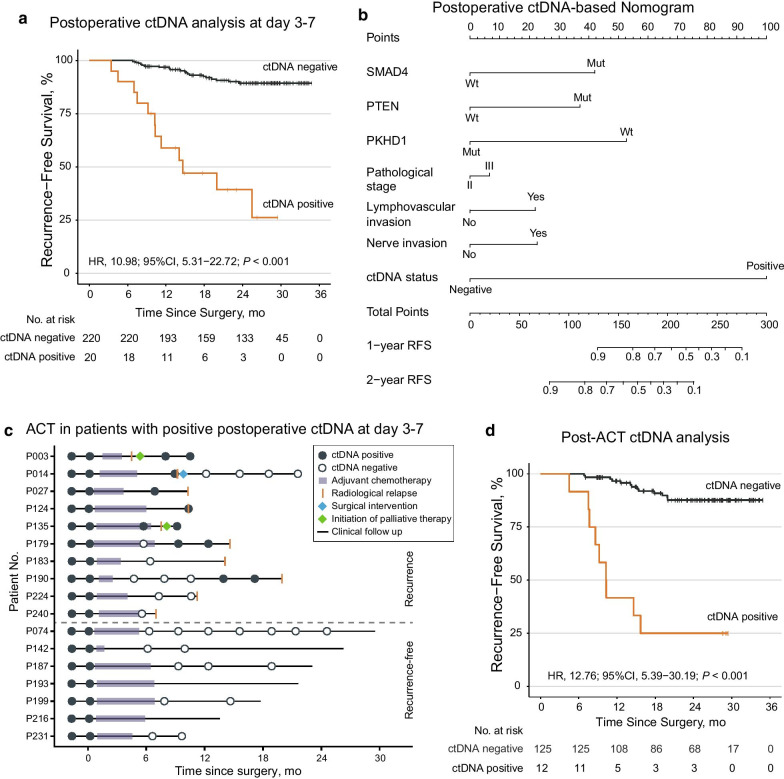
Association of postoperative and post-adjuvant chemotherapy ctDNA status with recurrence risk. **a** Kaplan–Meier estimates of recurrence-free survival (RFS) according to ctDNA status at day 3–7 postoperatively. **b** The nomogram constructed by selected clinicopathological factors, recurrent-mutated genes, and ctDNA status at day 3–7 postoperatively for predicting 1-year and 2-year RFS. Mut, mutant; Wt, wild-type. **c** The clinical courses together with ctDNA statuses of 17 out of 20 ctDNA-positive (at day 3–7 postoperation) patients who received adjuvant chemotherapy (ACT). **d** Kaplan–Meier curves of RFS of the 137 patients who had plasma samples drawn after ACT, stratified by ctDNA status at first sampling point post-ACT

To evaluate whether ctDNA status could reflect the existence of MRD and thus lead to disease recurrence, postoperative plasma samples at day 3–7 (median, day 5) were collected before hospital discharge and analyzed. The plasma samples were available for all the 240 patients, of which 20 (8.3%) were classified as ctDNA-positive, while 220 (91.7%) were ctDNA-negative. Low recurrence risk was observed in ctDNA-negative patients, with a 2-year RFS rate of 89.4% [95% CI 85.1–93.9%]. On the contrary, ctDNA-positive patients had extremely high recurrence risk compared with that of ctDNA-negative patients (HR, 10.98; 95% CI 5.31–22.72; *P* < 0.001; Fig. 2a), with a 2-year RFS rate of 39.3% [95% CI 21.5–71.8%]. In detail, among 20 ctDNA-positive patients, 60% (12/20) experienced radiological relapse. In terms of the other 8 patients without documented recurrence, 7 of them received ACT and their ctDNA status turned negative afterward, or they were lost to follow-up rather earlier (Additional file 12: Figure S4).

Univariate Cox regression analysis of known clinicopathological risk factors showed that pathological stage, lymphovascular invasion, nerve invasion, and ctDNA status at day 3–7 postoperation were all significantly associated with RFS; whereas the later multivariate analysis revealed that ctDNA status at day 3–7 postoperation was the most significant prognostic factor even after adjusted for preoperative ctDNA status (Table 1). Using LASSO Cox regression analysis (see Additional file 3: Methods), we further identified three recurrent-mutated genes whose somatic mutational status in the primary tumor was significantly associated with RFS, namely SMAD4, PTEN and PKHD1. We thus combined these risk factors and successfully constructed a nomogram for RFS prediction (Fig. 2b). Sensitivity analysis showed that the inclusion of ctDNA status at day 3–7 postoperation in the nomogram model could significantly improve its discrimination power, as the Harrell’s C-index was 0.802 [95% CI 0.727–0.882] with ctDNA status versus 0.716 [95% CI 0.630–0.816] without ctDNA status (*P* < 0.001).

**Table 1:** Univariate and multivariate Cox analysis of recurrence-free survival by clinicopathological variables and ctDNA status at 3–7 days postoperation

Variable	Univariate analysis		Multivariate analysis	
	HR (95% CI)	P*	HR (95% CI)	P*
*Age, years*				
≤ 60 versus > 60	1.44 (0.71–2.91)	0.313		
*Sex*				
Male versus female	0.74 (0.37–1.47)	0.387		
*Primary tumor location*				
Right-sided versus left-sided	1.87 (0.93–3.74)	0.077		
*Pathological stage*				
III versus II	2.26 (1.07–4.79)	**0.032**	1.18 (0.50–2.77)	0.706
*Lymphovascular invasion*				
Yes versus no	2.60 (1.30–5.22)	**0.007**	1.53 (0.67–3.48)	0.316
*Nerve invasion*				
Yes versus no	2.04 (1.02–4.08)	**0.045**	1.99 (0.90–4.39)	0.091
*Histological type*				
Mucinous versus adenocarcinoma	0.90 (0.27–2.94)	0.856		
*Histological grade*				
Poor versus medium/well	0.88 (0.38–2.03)	0.757		
*MSI status*				
MSI-L/MSS versus MSI-H	0.99 (0.30–3.24)	0.984		
*Preoperative ctDNA status*				
Positive versus negative	5.66 (1.72–18.57)	**0.004**	3.79 (1.12–12.86)	**0.032**
*ctDNA status at day 3–7 postoperation*				
Positive versus negative	10.98 (5.31–22.72)	**< 0.001**	8.02 (3.59–17.92)	**< 0.001**

*P value in bold denotes statistically significant

Subgroup analysis showed that ctDNA status was significantly associated with RFS in both the ACT subgroup and the non-ACT subgroup, indicating that the association between ctDNA status and RFS was independent of ACT (Additional file 13: Figure S5). Among 174 patients receiving ACT, ctDNA positivity was still associated with a high risk of recurrence (HR 9.99; 95% CI 4.40–22.69; *P* < 0.001). On the contrary, the 2-year RFS rate of ctDNA-negative patients who received ACT was 89.6% [95% CI 84.5–95.0%], which was similar to that of ctDNA-negative patients who did not receive ACT (89.2% [95% CI 81.4–97.8%]).

### Dynamic ctDNA change reflected the efficacy of ACT

To investigate whether the dynamic change of ctDNA could reflect the eradication of MRD by ACT, we analyzed the concordance between the clinical courses and the ctDNA statuses of 17 out of 20 ctDNA-positive (at day 3–7 postoperation) patients who received ACT (Fig. 2c).

Of these 17 patients, 10 patients experienced radiological relapse while 7 patients remained recurrence-free at the last follow-up, supporting the notion that some patients with high relapse risk can benefit from ACT. Notably, five recurrence-free patients with available serial plasma samples showed complete ctDNA clearance after ACT and remained negative during the following surveillance. In contrast, of the other 10 patients who experienced recurrence, seven remained ctDNA-positive consistently or regained ctDNA positivity after a temporary ctDNA clearance by ACT. These results showed that ctDNA status shifting was generally in good concordance with patients’ clinical courses, except for three patients, i.e., P183, P224 and P240, who still relapsed after ctDNA clearance by ACT (Fig. 2c), albeit the ctDNA status of P183 might regain positivity in a ~ 8-month interval between the last ctDNA sampling and disease recurrence as we observed in P190.

### ctDNA status after ACT significantly associated with recurrence risk

Post-ACT risk stratification and management are also critical, but indicators for such decision-making are still lacking. Among 137 patients had plasma samples after ACT, 125 patients were ctDNA-negative while 12 patients were ctDNA-positive at the first sampling point after ACT. Significantly reduced RFS of ctDNA-positive patients was observed compared with that of their counterparts (HR 12.76; 95% CI 5.39–30.19; *P* < 0.001; Fig. 2d), and the 2-year RFS rates were 25.0% [95% CI 9.4–66.6%] for ctDNA-positive patients versus 87.7% [95% CI 81.5–94.2%] for ctDNA-negative patients. Univariate and multivariate Cox regression analysis showed that ctDNA status at the first sampling point after ACT was still the most significant prognostic factor associated with recurrence risk (Additional file 14: Table S5).

### Serial ctDNA status significantly associated with clinical outcomes

**Fig. 3:**
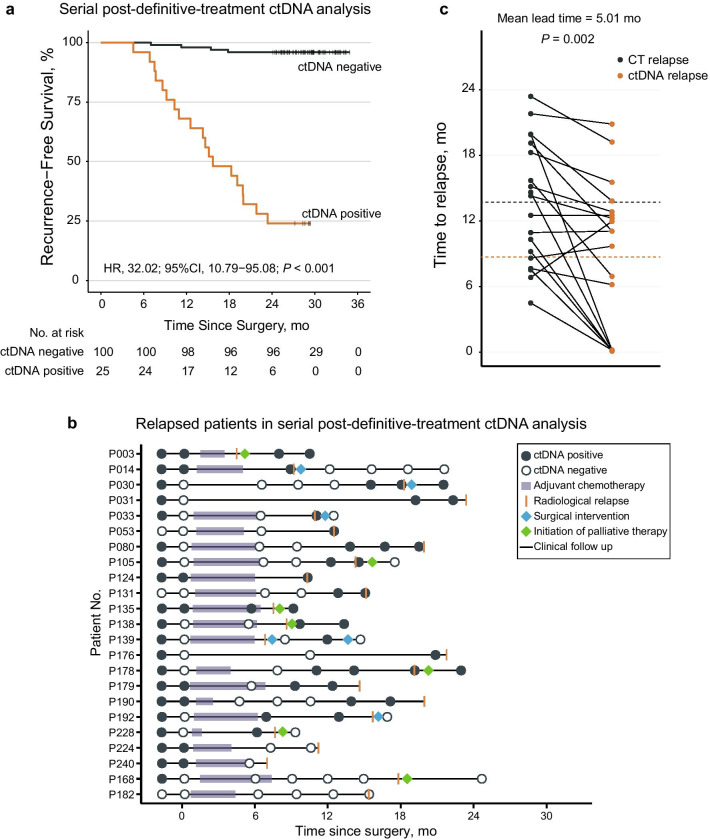
Association of ctDNA analyses with early detection of recurrence. **a** Kaplan–Meier curves of recurrence-free survival of the 125 patients who were included in the serial post-definitive treatment analysis, stratified by serial ctDNA status. **b** ctDNA profiling results and the corresponding clinical courses of the 23 relapsed patients included in the serial post-definitive treatment analysis. **c** Comparison of time from surgery to disease recurrence by ctDNA and computed tomography (CT) scanning, dashed lines indicate mean time of recurrence based on CT (13.70 months) and ctDNA (8.69 months). (*N* = 19, Student’s t test)

To evaluate the role of ctDNA in recurrence surveillance after definitive therapy, we restricted our analysis in patients with serial plasma samples (≥ 3 samples postoperatively or with sample(s) drawn within 3 months around an early relapse) and sufficient follow-up durations (≥ 24 months or relapse), which gave rise to a serial ctDNA analysis subgroup of 125 patients. Patients were classified as serial ctDNA-positive if one or more post-definitive treatment plasma samples were ctDNA-positive. Remarkably, the serial ctDNA-positive patients had extremely high recurrence risk (2-year RFS rate, 24.0% [95% CI 11.9–48.2%]) while only 4 out of 100 serial ctDNA-negative patients experienced recurrence (2-year RFS rate, 96.0% [95% CI 92.2–99.9%]) (Fig. 3a, HR 32.02; 95% CI 10.79–95.08; *P* < 0.001). Univariate and multivariate Cox regression analysis also revealed that serial ctDNA status was still the most significant risk factor (Additional file 15: Table S6).

The serial ctDNA profiling results and clinical courses of 23 patients who experienced recurrence are presented in Fig. 3b, of which 19 patients were identified as serial ctDNA-positive. While in 102 patients who did not relapse, 96 of them were identified as serial ctDNA-negative (Additional file 16: Figure S6), and the other six patients presented with transient ctDNA positivity that turned into and remained negative throughout follow-up, which could be some unappreciated technical artifacts that lead to false positive. Taken together, the sensitivity was 82.6% and the specificity was 94.1%, with an overall accuracy in identifying disease recurrence of 92.0%.

Furthermore, ctDNA outperformed CEA in the early detection of recurrence. The mean lead time from ctDNA detection to imaging-confirmed recurrence was 5.01 months (*P* = 0.002; Fig. 3c), while CEA showed no significant lead time in recurrence detection (*P* = 0.199; Additional file 17: Figure S7). Notably, in nine patients with ≥ 2 ctDNA-positive plasma samples after definite treatment, an increase in the mean ctDNA variant allele frequency (VAF) was generally observed (Additional file 18: Figure S8), which was in accordance with the natural and gradual development of MRD when the patients awaited radiologic confirmation of recurrence. Thus, early detection of recurrence by ctDNA in surveillance might leverage the opportunity for early intervention and even secondary curative resection.

## Discussion

To the best of our knowledge, this prospective, observational, and multicenter study is by far the largest one to investigate the relationship between postoperative ctDNA status and recurrence risk in stage II/III CRC patients. The results from 240 patients strongly support the notion that positive ctDNA status could reflect the existence of MRD and thus the extremely high risk of disease recurrence. Across the whole clinical course, ctDNA status remained the most significant and independent predictor of RFS among all clinicopathological risk factors though the multivariate analysis may be not powered given the small subgroup size of ctDNA-positive patients, and the dynamic change of ctDNA status was in good concordance with clinical courses and outcomes. Consistent with recent studies addressing similar questions [14–17], our present study further confirmed the promising potential of ctDNA analysis in MRD detection after surgery and thus may guide the initiation and intensity/duration of ACT for CRC patients. Additionally, the early detection of residual diseases may provide patients with more chances to receive secondary curative surgery.

It’s groundbreaking that, not like any other conventional factors for recurrence risk stratification (high versus low), detection of ctDNA is the reflection of MRD itself (yes versus no). As the ctDNA measuring technology continues to evolve and manifest its significance [19], the real-time detection of MRD using ctDNA may help guide the precise administration of ACT, evaluation of ACT efficacy, and monitoring of disease recurrence. The potential paradigm-changing clinical applications of this ctDNA-guided strategy can be summarized as follows. Patients with positive ctDNA after surgery may be subjected to ACT to eradicate the MRD regardless the status of other clinicopathological risk factors and monitored by ctDNA during ACT for efficacy assessment. For patients who remain ctDNA-positive after standard-of-care ACT courses, escalation of ACT or change of regimens may be considered according to potential actionable targets revealed by ctDNA. Besides, as ctDNA showed significant lead time to standard-of-care CT scan, whose optimal frequency does not reach common consensus yet [20], intensified frequency of CT scan or PET-CT guided by ctDNA may be adopted to identify the tumor lesions earlier and increase the possibility of a secondary curative intervention. While for patients with negative ctDNA after surgery, even with stage III disease, ACT may be withheld with ctDNA monitoring offered and ACT administrated once ctDNA turns to positive afterward. As more than half of the stage III CRC patients could be cured by surgery alone [21], this ctDNA-guided strategy may benefit patients by sparing them from unnecessary drug toxicity, economic burden, and even radiological exposure. Of note, a small proportion of patients who were ctDNA-negative at day 3–7/day 30 postoperatively still relapsed (9.1% in this study and 11.9% reported by Reinert et al. [17]). Thus, although our study underscores the utility of postoperative ctDNA surveillance, randomized clinical trials (RCTs) are necessary to further determine to what extent the deferred ACT may compromise the survival benefit of these patients. RCTs, such as the Australian DYNAMIC/DYNAMIC-III trials [22, 23], the French CIRCULATE trial [24], and the US COBRA trial [25], are now being conducted to compare the above-mentioned ctDNA-guided strategy with the standard of care. It should be emphasized that neither the results of our study nor those of other previous researches could directly lead to the transitioning of the ctDNA-guided administration of postoperative ACT and disease surveillance into real-world clinical practice until the survival benefit of this strategy over the current standard of care is confirmed by RCTs, and one should also be cautious about “lead time bias” when drawing conclusions from the results of RCTs using RFS as primary endpoint.

Our comprehensive NGS panel-based ctDNA detecting strategy also has advantages over the customized multiplex PCR-based NGS approach in several aspects. Two representative ctDNA detecting technologies were employed in the previous reports of ctDNA analysis in resectable CRC, namely the Safe-SeqS method and the Signatera RUO workflow [14–17]. These two technologies both adopt a highly customized strategy of detecting one or several patient-specific somatic variants, which allows the employment of ultra-deep sequencing to achieve high sensitivity and specificity [26]. However, the relatively long turnaround duration may limit its ability to provide timely ctDNA results before ACT, which should be commenced no later than 6–8 weeks after surgery [9]. On the contrary, the strategy of comprehensive NGS panel testing on both tumor and plasma samples saves us much time by avoiding the primary tumor-based and time-consuming step of patient-specific PCR primer pair(s) design and synthesis for ctDNA detection. Indeed, the first postoperative ctDNA evaluation in previous reports usually started at 4–10 weeks after surgery [14–17], while our ctDNA evaluation performed as early as 3–7 days postoperatively was also able to identify patients with remarkably high recurrence risk. Moreover, the detection ability of the multiplex PCR-based NGS strategy spanning the entire clinical course may be restricted by its customization for one or several predefined patient-specific variants. While more patient-specific somatic variants and even new variants could be considered and captured as a large number of genomic regions were covered by our NGS panel, which might also mitigate the impact of heterogeneity of the primary tumor and clonal evolution under selection pressures from the immune system or ACT [27]. Besides, the information conveyed by our test may also help in identifying the resistance mechanisms to ACT and potential actionable targets for subsequent treatments [28].

Our ctDNA analyses identified recurrence with an overall accuracy of 92.0% and a sensitivity of 82.6% during surveillance, which is comparable to the reported sensitivity of 88% by Reinert et al. using ultra-deep sequencing [17]. Nevertheless, as increasing evidence reveals that the VAF of ctDNA in the plasma could be as low as ~ 0.01%[29], we acknowledged that a mean sequencing depth of ~ 4000 × achieved in this study may not detect the ctDNA with a rather low VAF. This compromised the sensitivity of our ctDNA detection and might lead to false negative when dealing with ultra-low concentration ctDNA. As shown in this study, though the preoperative detection rate of ctDNA was much higher than the elevated rate of CEA (64.2% vs 39.4%), it was still lower than those reported in two previous studies, which were 76.7% in locally advanced rectal cancer [30] and 88.5% in resectable CRC [17]. Therefore, we are dedicated to modifying our technology to further improve the sensitivity. Based on our whole-exome sequencing database containing > 1000 CRC samples (unpublished data), we continue to optimize the targeted genomic regions from pan cancer-related genes to more CRC-specific ones. Meanwhile, our future sequencing depth can reach > 30,000 × to ensure the detection of ctDNA with rather low VAF [31].

## Conclusion

In conclusion, this large-scale, prospective, observational, and multicenter study suggests that serial detection of ctDNA could reflect the existence of MRD and identify disease recurrence ahead of radiological imaging. ctDNA evaluation, as early as 3–7 days postoperatively, may facilitate risk stratification and decision-making in the postsurgical management of patients with stage II/III CRC. Further interventional RCTs are warranted.

## Supplementary Information

**Additional file 1: Table S1.** Detailed clinicopathological information of 240 evaluable patients.

**Additional file 2: Table S2.** Gene list of the Geneseeq Prime™ 425-gene panel.

**Additional file 3: Methods.**

**Additional file 4: Table S3.** Baseline characteristics of 240 evaluable patients.

**Additional file 5: Figure S1.** Heatmap demonstrating the mutational landscape of the primary tumors from 240 evaluable patients, with the upper third depicting the mutation counts of each patient, the middle third presenting the top 20 recurrent-mutated genes in this cohort, and the lower third showing the corresponding clinicopathological features.

**Additional file 6: Dataset S1.** List of somatic variants in the primary tumors of 240 patients.

**Additional file 7: Dataset S2.** Serial ctDNA profiling results of 240 evaluable patients.

**Additional file 8: Figure S2.** Preoperative examination of CEA and ctDNA in 240 evaluable patients stratified by pathological stage. (Nine patients with no CEA information available pre-operation).

**Additional file 9: Dataset S3.** Serial serum CEA results.

**Additional file 10: Figure S3.** Kaplan–Meier estimates of recurrence-free survival (RFS) according to preoperative CEA and ctDNA status.

**Additional file 11: Table S4.** Patients’ clinical features stratified by preoperative ctDNA status.

**Additional file 12: Figure S4.** The clinical courses together with ctDNA statuses of 8 out of 20 ctDNA-positive (at day 3–7 postoperation) patients without documented recurrence.

**Additional file 13: Figure S5.** Subgroup analysis of the association between ctDNA status and recurrence-free survival in patients receiving adjuvant chemotherapy or not.

**Additional file 14: Table S5.** Univariate and multivariable Cox analysis of recurrence-free survival by clinicopathological variables and ctDNA status at first sampling point after adjuvant chemotherapy.

**Additional file 15: Table S6.** Univariate and multivariable Cox analysis of recurrence-free survival by clinicopathological variables and ctDNA status in surveillance samples.

**Additional file 16: Figure S6.** The clinical courses together with ctDNA statuses of 102 censored patients included in serial post-definitive treatment ctDNA analysis.

**Additional file 17: Figure S7.** Comparison of time from surgery to disease recurrence by CEA and computed tomography (CT) scanning, dashed lines indicate mean time of recurrence based on CT (13.46 months) and CEA (12.08 months). (*N*=14, Student’s t-test)

**Additional file 18: Figure S8.** In 9 patients with ≥2 ctDNA-positive plasma samples after definite treatment, the mean variant allele frequency (VAF) of their ctDNA in the plasma was demonstrated from first ctDNA detection to radiological confirmation of relapse (early time points before and during adjuvant chemotherapy were omitted). Each colored curve indicates data of one patient.

## Data Availability

All relevant data are available in the supplementary materials or on request from the corresponding author for reuse under research purpose only.

## Abbreviations

ACT: Adjuvant chemotherapy
CEA: Carcinoembryonic antigen
cfDNA: Circulating free DNA
CI: Confidence interval
CRC: Colorectal cancer
CT: Computed tomography
ctDNA: Circulating tumor DNA
HR: Hazard ratio
MRD: Minimal residual disease
MSI-H: High microsatellite instability
MSI-L: Low microsatellite instability
MSS: Microsatellite stable
NGS: Next-generation sequencing
RCTs: Randomized clinical trials
RFS: Recurrence-free survival
VAF: Variant allele frequency
