# Supplementary material for: Postoperative circulating tumor DNA as markers of recurrence risk in stages II to III colorectal cancer
Source: J Hematol Oncol. 2021 May 17;14:80. doi: 10.1186/s13045-021-01089-z (PMC8130394; doi:10.1186/s13045-021-01089-z)
Supplement: Supplementary file 1 — Additional file 1: Table S1. Detailed clinicopathological information of 240 evaluable patients. [file 13045_2021_1089_MOESM1_ESM.docx]

| **Table S1. Detailed clinicopathological information of 240 evaluable patients.** | | | | | | | | | | | | |
| --- | --- | --- | --- | --- | --- | --- | --- | --- | --- | --- | --- | --- |
| **Patient No.** | **Age** | **Sex** | **PTL^a^** | **Stage** | **Hist_type^b^** | **Hist_grade^c^** | **LV_invasion^d^** | **N_invasion^e^** | **MSI** | **ACT^f^** | **R_time^g^** | **R_treatment^h^** |
| P001 | 54 | Male | Right | III | Adeno | poor | no | yes | MSI_H | yes |  |  |
| P002 | 68 | Male | Right | III | Adeno | medium/well | no | yes | MSS | yes |  |  |
| P003 | 58 | Female | Right | III | Mucinous | poor | yes | yes | MSS | yes | 4.50 | Palliative |
| P004 | 65 | Male | Left | III | Adeno | medium/well | yes | yes | MSS | yes |  |  |
| P005 | 54 | Male | Right | III | Adeno | poor | no | yes | MSS | yes |  |  |
| P006 | 64 | Male | Right | II | Adeno | medium/well | no | yes | MSI_H | no | 12.65 | Palliative |
| P007 | 48 | Male | Left | III | Mucinous | poor | no | no | MSS | yes |  |  |
| P008 | 39 | Female | Right | II | Adeno | poor | no | no | MSI_H | no |  |  |
| P009 | 49 | Male | Left | III | Adeno | medium/well | yes | yes | MSS | yes |  |  |
| P010 | 55 | Male | Right | II | Adeno | medium/well | no | no | MSS | no |  |  |
| P011 | 69 | Male | Left | III | Mucinous | poor | yes | no | MSS | yes |  |  |
| P012 | 73 | Male | Left | II | Adeno | medium/well | no | yes | MSS | yes |  |  |
| P013 | 64 | Female | Right | II | Adeno | medium/well | no | no | MSS | no |  |  |
| P014 | 64 | Male | Right | II | Adeno | poor | no | no | MSS | yes | 9.20 | Surgery |
| P015 | 53 | Male | Left | II | Adeno | medium/well | no | no | MSS | no |  |  |
| P016 | 45 | Male | Right | II | Mucinous | poor | no | no | MSI_H | no |  |  |
| P017 | 64 | Female | Right | II | Adeno | medium/well | no | no | MSS | no |  |  |
| P018 | 53 | Female | Left | III | Adeno | poor | yes | yes | MSS | yes |  |  |
| P019 | 54 | Male | Left | II | Adeno | medium/well | no | no | MSS | yes |  |  |
| P020 | 74 | Male | Left | II | Adeno | medium/well | no | no | MSS | no |  |  |
| P021 | 64 | Male | Right | II | Adeno | poor | yes | yes | MSS | yes |  |  |
| P022 | 47 | Male | Left | II | Adeno | medium/well | yes | yes | MSS | yes |  |  |
| P023 | 61 | Female | Left | II | Adeno | medium/well | no | no | MSS | yes |  |  |
| P024 | 52 | Male | Right | III | Adeno | medium/well | yes | yes | MSS | yes |  |  |
| P025 | 54 | Male | Left | II | Adeno | medium/well | no | no | MSS | no |  |  |
| P026 | 48 | Female | Left | II | Adeno | medium/well | yes | yes | MSS | yes |  |  |
| P027 | 44 | Male | Left | III | Adeno | medium/well | yes | yes | MSS | yes | 10.28 | Palliative |
| P028 | 49 | Female | Right | II | Adeno | poor | no | yes | MSS | no |  |  |
| P029 | 66 | Female | Left | III | Adeno | medium/well | no | yes | MSS | yes |  |  |
| P030 | 65 | Male | Right | II | Adeno | medium/well | no | no | MSS | no | 18.27 | Surgery |
| P031 | 79 | Female | Right | II | Adeno | medium/well | no | no | MSS | no | 23.39 | Palliative |
| P032 | 73 | Male | Left | II | Adeno | medium/well | no | no | MSS | no |  |  |
| P033 | 49 | Female | Right | III | Adeno | medium/well | no | yes | MSS | yes | 10.94 | Surgery |
| P034 | 64 | Male | Left | II | Adeno | medium/well | no | no | MSS | yes |  |  |
| P035 | 57 | Male | Left | II | Adeno | medium/well | no | no | MSS | no | 25.49 | Palliative |
| P036 | 59 | Female | Left | II | Adeno | medium/well | yes | no | MSS | yes |  |  |
| P037 | 63 | Female | Left | II | Adeno | poor | no | yes | MSS | yes |  |  |
| P038 | 60 | Male | Left | II | Adeno | medium/well | no | no | MSS | no |  |  |
| P039 | 70 | Female | Right | III | Adeno | medium/well | yes | yes | MSS | yes |  |  |
| P040 | 76 | Male | Left | II | Adeno | medium/well | yes | yes | MSS | no |  |  |
| P041 | 64 | Male | Left | II | Adeno | medium/well | yes | yes | MSS | yes |  |  |
| P042 | 64 | Female | Right | II | Mucinous | poor | no | no | MSS | no |  |  |
| P043 | 61 | Female | Left | II | Adeno | medium/well | no | no | MSS | no |  |  |
| P044 | 69 | Male | Left | III | Adeno | medium/well | no | no | MSS | yes |  |  |
| P045 | 49 | Female | Right | III | Adeno | medium/well | no | no | MSS | yes |  |  |
| P046 | 64 | Male | Left | III | Adeno | poor | yes | yes | MSS | yes |  |  |
| P047 | 31 | Female | Left | III | Adeno | medium/well | yes | no | MSS | yes |  |  |
| P048 | 75 | Female | Right | II | Adeno | medium/well | no | no | MSS | no |  |  |
| P049 | 73 | Female | Left | II | Adeno | medium/well | no | no | MSS | no |  |  |
| P050 | 46 | Female | Right | II | Adeno | medium/well | no | no | MSS | no | 3.42 | Palliative |
| P051 | 61 | Female | Left | III | Adeno | medium/well | no | no | MSS | yes |  |  |
| P052 | 46 | Male | Left | III | Adeno | medium/well | no | yes | MSS | no |  |  |
| P053 | 58 | Female | Left | III | Adeno | medium/well | yes | yes | MSS | yes | 12.52 | Palliative |
| P054 | 47 | Female | Right | II | Adeno | medium/well | no | yes | MSS | no |  |  |
| P055 | 58 | Male | Left | II | Adeno | medium/well | yes | no | MSS | yes |  |  |
| P056 | 76 | Female | Right | II | Adeno | medium/well | no | no | MSS | no |  |  |
| P057 | 52 | Male | Left | III | Adeno | medium/well | yes | yes | MSS | yes |  |  |
| P058 | 64 | Female | Left | III | Adeno | medium/well | no | yes | MSS | yes |  |  |
| P059 | 52 | Male | Left | III | Adeno | poor | no | yes | MSS | yes |  |  |
| P060 | 69 | Female | Right | III | Adeno | poor | yes | no | MSI_H | yes |  |  |
| P061 | 49 | Female | Left | II | Adeno | medium/well | no | no | MSS | no |  |  |
| P062 | 64 | Female | Right | II | Adeno | medium/well | no | no | MSS | yes |  |  |
| P063 | 65 | Male | Left | II | Adeno | medium/well | no | yes | MSS | yes |  |  |
| P064 | 69 | Female | Right | III | Mucinous | poor | no | no | MSS | yes |  |  |
| P065 | 56 | Female | Left | III | Adeno | medium/well | yes | no | MSS | yes |  |  |
| P066 | 56 | Male | Left | II | Adeno | medium/well | yes | yes | MSS | yes |  |  |
| P067 | 77 | Male | Right | III | Adeno | medium/well | no | yes | MSS | yes |  |  |
| P068 | 63 | Male | Left | II | Adeno | medium/well | no | no | MSS | yes |  |  |
| P069 | 70 | Male | Left | III | Adeno | poor | yes | yes | MSS | yes |  |  |
| P070 | 60 | Male | Left | III | Adeno | medium/well | no | no | MSS | yes |  |  |
| P071 | 66 | Female | Left | II | Adeno | medium/well | no | no | MSS | no |  |  |
| P072 | 65 | Male | Left | II | Adeno | medium/well | no | yes | MSS | yes | 7.16 | Palliative |
| P073 | 63 | Male | Right | III | Adeno | poor | no | no | MSS | yes |  |  |
| P074 | 63 | Female | Left | III | Mucinous | poor | yes | no | MSS | yes |  |  |
| P075 | 54 | Male | Left | II | Adeno | medium/well | yes | no | MSS | yes |  |  |
| P076 | 64 | Male | Left | III | Adeno | medium/well | yes | no | MSS | yes |  |  |
| P077 | 54 | Female | Left | III | Adeno | medium/well | yes | yes | MSS | yes |  |  |
| P078 | 78 | Female | Right | II | Mucinous | poor | yes | yes | MSI_H | no | 8.28 | Surgery |
| P079 | 59 | Female | Left | II | Adeno | medium/well | no | yes | MSS | yes |  |  |
| P080 | 31 | Female | Right | III | Adeno | poor | yes | no | MSS | yes | 19.91 | Surgery |
| P081 | 77 | Male | Left | II | Adeno | medium/well | no | no | MSS | no |  |  |
| P082 | 54 | Male | Left | II | Adeno | medium/well | no | no | MSS | no |  |  |
| P083 | 62 | Male | Left | III | Adeno | poor | yes | yes | MSS | yes |  |  |
| P084 | 78 | Female | Left | II | Adeno | medium/well | no | no | MSS | yes |  |  |
| P085 | 58 | Female | Left | II | Mucinous | poor | yes | no | MSS | yes |  |  |
| P086 | 58 | Male | Left | II | Adeno | medium/well | no | no | MSS | yes |  |  |
| P087 | 50 | Female | Left | II | Adeno | medium/well | no | no | MSS | yes |  |  |
| P088 | 54 | Female | Left | III | Adeno | medium/well | no | yes | MSS | yes |  |  |
| P089 | 49 | Female | Left | II | Adeno | medium/well | no | yes | MSS | yes |  |  |
| P090 | 69 | Female | Right | II | Adeno | medium/well | no | no | MSS | no |  |  |
| P091 | 62 | Female | Right | II | Adeno | medium/well | yes | no | MSS | no |  |  |
| P092 | 54 | Female | Left | III | Adeno | medium/well | yes | yes | MSS | yes |  |  |
| P093 | 66 | Male | Left | II | Adeno | medium/well | no | yes | MSS | no |  |  |
| P094 | 75 | Male | Left | II | Adeno | medium/well | no | no | MSS | no |  |  |
| P095 | 59 | Male | Left | III | Adeno | medium/well | no | yes | MSS | yes |  |  |
| P096 | 35 | Female | Right | II | Adeno | poor | yes | yes | MSS | yes |  |  |
| P097 | 30 | Female | Left | III | Adeno | medium/well | no | yes | MSS | yes |  |  |
| P098 | 72 | Male | Right | II | Adeno | medium/well | yes | yes | MSS | no |  |  |
| P099 | 63 | Female | Left | II | Adeno | medium/well | no | no | MSS | yes |  |  |
| P100 | 73 | Male | Right | II | Adeno | medium/well | yes | yes | MSS | yes |  |  |
| P101 | 76 | Male | Left | III | Adeno | medium/well | no | no | MSS | yes |  |  |
| P102 | 67 | Male | Right | III | Mucinous | poor | yes | yes | MSS | yes |  |  |
| P103 | 51 | Male | Right | II | Adeno | medium/well | yes | yes | MSS | yes |  |  |
| P104 | 66 | Male | Left | II | Adeno | medium/well | no | no | MSS | no |  |  |
| P105 | 53 | Male | Left | III | Adeno | medium/well | yes | yes | MSS | yes | 14.29 | Palliative |
| P106 | 56 | Male | Left | II | Adeno | medium/well | no | no | MSI_H | no |  |  |
| P107 | 63 | Male | Left | III | Adeno | poor | no | no | MSS | yes |  |  |
| P108 | 61 | Male | Left | III | Adeno | medium/well | no | no | MSS | yes |  |  |
| P109 | 73 | Male | Left | II | Adeno | medium/well | no | no | MSS | yes |  |  |
| P110 | 56 | Female | Left | III | Adeno | medium/well | yes | no | MSS | yes |  |  |
| P111 | 70 | Male | Left | II | Adeno | medium/well | no | no | MSS | yes |  |  |
| P112 | 39 | Male | Right | II | Adeno | medium/well | no | no | MSS | no |  |  |
| P113 | 51 | Female | Left | III | Adeno | medium/well | no | yes | MSS | yes |  |  |
| P114 | 78 | Female | Left | III | Adeno | medium/well | yes | yes | MSS | no |  |  |
| P115 | 65 | Female | Left | II | Adeno | medium/well | no | no | MSS | no |  |  |
| P116 | 39 | Female | Left | III | Adeno | medium/well | yes | no | MSS | yes |  |  |
| P117 | 51 | Male | Left | III | Adeno | poor | yes | no | MSS | yes |  |  |
| P118 | 64 | Female | Right | II | Adeno | medium/well | no | no | MSS | no |  |  |
| P119 | 63 | Female | Right | III | Adeno | medium/well | no | yes | MSS | yes |  |  |
| P120 | 56 | Female | Left | III | Mucinous | poor | no | no | MSS | yes |  |  |
| P121 | 50 | Female | Left | III | Adeno | medium/well | no | no | MSS | yes |  |  |
| P122 | 80 | Male | Right | II | Mucinous | poor | no | no | MSI_H | no |  |  |
| P123 | 67 | Female | Left | II | Mucinous | poor | no | yes | MSI_H | no |  |  |
| P124 | 64 | Female | Right | III | Adeno | medium/well | yes | yes | MSS | yes | 10.32 | Palliative |
| P125 | 61 | Female | Left | II | Adeno | medium/well | no | no | MSS | yes |  |  |
| P126 | 49 | Male | Right | II | Mucinous | poor | no | no | MSS | yes |  |  |
| P127 | 69 | Male | Right | II | Adeno | medium/well | no | no | MSS | no |  |  |
| P128 | 72 | Female | Right | II | Mucinous | poor | no | no | MSS | no |  |  |
| P129 | 73 | Male | Right | II | Adeno | medium/well | no | no | MSS | yes |  |  |
| P130 | 61 | Male | Left | III | Adeno | medium/well | no | no | MSS | yes |  |  |
| P131 | 62 | Male | Right | III | Adeno | medium/well | yes | no | MSS | yes | 15.15 | Surgery |
| P132 | 54 | Female | Left | II | Adeno | medium/well | no | no | MSS | yes |  |  |
| P133 | 66 | Male | Right | II | Adeno | medium/well | no | no | MSI_H | no |  |  |
| P134 | 63 | Male | Right | III | Mucinous | poor | yes | yes | MSS | yes |  |  |
| P135 | 44 | Female | Left | III | Adeno | medium/well | no | no | MSS | yes | 7.52 | Palliative |
| P136 | 19 | Female | Right | III | Mucinous | poor | yes | yes | MSS | yes |  |  |
| P137 | 35 | Male | Left | II | Mucinous | poor | no | yes | MSS | no |  |  |
| P138 | 52 | Female | Left | III | Adeno | medium/well | yes | yes | MSS | yes | 8.61 | Palliative |
| P139 | 36 | Female | Left | III | Mucinous | poor | yes | yes | MSS | yes | 6.83 | Surgery |
| P140 | 64 | Female | Left | III | Adeno | poor | no | no | MSS | yes |  |  |
| P141 | 49 | Male | Left | III | Adeno | medium/well | yes | yes | MSS | yes |  |  |
| P142 | 66 | Female | Right | II | Adeno | medium/well | no | no | MSS | yes |  |  |
| P143 | 59 | Female | Right | III | Adeno | medium/well | no | no | MSS | yes |  |  |
| P144 | 58 | Male | Left | II | Adeno | medium/well | yes | no | MSS | yes |  |  |
| P145 | 55 | Male | Left | II | Adeno | medium/well | no | no | MSS | yes |  |  |
| P146 | 37 | Male | Right | II | Adeno | medium/well | no | no | MSI_H | no |  |  |
| P147 | 84 | Female | Left | II | Adeno | medium/well | no | no | MSS | no |  |  |
| P148 | 56 | Female | Left | III | Adeno | medium/well | no | no | MSS | yes |  |  |
| P149 | 48 | Male | Left | II | Adeno | medium/well | no | no | MSS | no |  |  |
| P150 | 63 | Female | Right | II | Adeno | medium/well | no | no | MSS | no | 14.98 | Surgery |
| P151 | 66 | Female | Right | III | Adeno | medium/well | no | no | MSS | yes |  |  |
| P152 | 59 | Female | Left | II | Adeno | medium/well | no | yes | MSS | yes |  |  |
| P153 | 55 | Female | Left | II | Adeno | medium/well | no | no | MSS | yes |  |  |
| P154 | 74 | Female | Right | III | Adeno | poor | no | no | MSS | yes |  |  |
| P155 | 71 | Female | Right | III | Mucinous | poor | yes | no | MSS | yes |  |  |
| P156 | 43 | Male | Left | III | Adeno | medium/well | yes | yes | MSS | yes |  |  |
| P157 | 70 | Male | Right | II | Adeno | medium/well | yes | yes | MSS | yes |  |  |
| P158 | 67 | Female | Right | II | Adeno | poor | no | no | MSI_H | no |  |  |
| P159 | 78 | Male | Right | II | Adeno | medium/well | no | no | MSS | yes |  |  |
| P160 | 49 | Female | Left | III | Adeno | medium/well | yes | yes | MSS | yes |  |  |
| P161 | 67 | Male | Left | III | Adeno | medium/well | yes | no | MSS | yes |  |  |
| P162 | 37 | Female | Left | III | Adeno | medium/well | yes | yes | MSS | yes |  |  |
| P163 | 60 | Male | Left | II | Adeno | medium/well | no | no | MSS | yes |  |  |
| P164 | 36 | Male | Right | III | Adeno | poor | yes | no | MSI_H | no |  |  |
| P165 | 46 | Male | Right | III | Adeno | medium/well | no | no | MSI_H | yes |  |  |
| P166 | 70 | Male | Left | III | Adeno | medium/well | yes | yes | MSS | no |  |  |
| P167 | 50 | Male | Left | III | Adeno | medium/well | no | yes | MSS | yes |  |  |
| P168 | 59 | Male | Left | III | Adeno | medium/well | yes | no | MSS | yes | 17.81 | Palliative |
| P169 | 65 | Male | Left | II | Adeno | medium/well | no | yes | MSS | yes |  |  |
| P170 | 56 | Male | Left | II | Adeno | medium/well | no | no | MSS | no |  |  |
| P171 | 51 | Male | Left | III | Adeno | medium/well | no | yes | MSS | yes |  |  |
| P172 | 56 | Male | Left | II | Adeno | medium/well | no | no | MSS | yes |  |  |
| P173 | 55 | Female | Left | III | Adeno | medium/well | yes | no | MSS | yes |  |  |
| P174 | 64 | Male | Left | III | Adeno | medium/well | no | yes | MSS | yes |  |  |
| P175 | 41 | Female | Right | II | Adeno | medium/well | no | no | MSS | no |  |  |
| P176 | 41 | Male | Right | II | Adeno | poor | no | no | MSI_H | no | 21.82 | Palliative |
| P177 | 55 | Male | Left | III | Adeno | medium/well | yes | yes | MSS | yes |  |  |
| P178 | 64 | Male | Left | III | Adeno | medium/well | yes | no | MSS | yes | 19.12 | Palliative |
| P179 | 35 | Female | Left | III | Adeno | medium/well | yes | yes | MSS | yes | 14.62 | Palliative |
| P180 | 55 | Male | Right | II | Adeno | medium/well | no | no | MSS | yes |  |  |
| P181 | 48 | Female | Left | II | Adeno | medium/well | no | no | MSS | yes |  |  |
| P182 | 58 | Male | Left | III | Adeno | medium/well | no | yes | MSS | yes | 15.38 | Palliative |
| P183 | 52 | Male | Right | III | Adeno | medium/well | yes | no | MSS | yes | 14.09 | Surgery |
| P184 | 58 | Male | Left | II | Adeno | medium/well | no | no | MSS | yes |  |  |
| P185 | 76 | Female | Left | III | Adeno | medium/well | no | no | MSS | yes |  |  |
| P186 | 58 | Male | Right | III | Adeno | medium/well | no | no | MSS | yes |  |  |
| P187 | 72 | Male | Left | III | Adeno | medium/well | no | no | MSS | yes |  |  |
| P188 | 60 | Female | Left | III | Adeno | poor | no | yes | MSS | no |  |  |
| P189 | 69 | Male | Right | III | Adeno | medium/well | yes | yes | MSS | yes |  |  |
| P190 | 70 | Female | Left | III | Adeno | medium/well | yes | yes | MSS | yes | 19.94 | Surgery |
| P191 | 56 | Male | Left | II | Adeno | poor | no | no | MSS | no |  |  |
| P192 | 44 | Male | Right | III | Adeno | medium/well | yes | yes | MSS | yes | 15.70 | Surgery |
| P193 | 65 | Male | Left | III | Adeno | medium/well | no | no | MSS | yes |  |  |
| P194 | 55 | Male | Left | III | Mucinous | poor | no | no | MSI_H | yes |  |  |
| P195 | 69 | Male | Left | III | Adeno | medium/well | yes | no | MSS | yes |  |  |
| P196 | 66 | Male | Left | II | Adeno | medium/well | no | no | MSS | yes |  |  |
| P197 | 42 | Male | Right | II | Adeno | medium/well | no | no | MSI_H | no |  |  |
| P198 | 66 | Male | Left | III | Adeno | poor | no | no | MSS | yes |  |  |
| P199 | 68 | Male | Right | III | Adeno | poor | yes | no | MSS | yes |  |  |
| P200 | 66 | Male | Left | III | Mucinous | poor | no | no | MSS | yes |  |  |
| P201 | 51 | Female | Left | II | Adeno | medium/well | no | no | MSS | yes |  |  |
| P202 | 61 | Male | Right | III | Mucinous | poor | no | no | MSS | yes |  |  |
| P203 | 57 | Male | Left | III | Adeno | medium/well | no | yes | MSS | yes |  |  |
| P204 | 62 | Male | Right | III | Mucinous | poor | no | no | MSI_H | yes |  |  |
| P205 | 46 | Male | Right | III | Adeno | medium/well | no | no | MSI_H | yes |  |  |
| P206 | 42 | Male | Right | II | Adeno | medium/well | no | no | MSS | yes |  |  |
| P207 | 51 | Male | Left | II | Adeno | medium/well | no | no | MSS | no |  |  |
| P208 | 71 | Male | Left | III | Adeno | medium/well | no | no | MSS | yes | 8.08 | Palliative |
| P209 | 43 | Female | Left | II | Adeno | medium/well | no | no | MSS | yes |  |  |
| P210 | 54 | Male | Left | II | Adeno | poor | no | no | MSS | no |  |  |
| P211 | 67 | Female | Right | II | Adeno | medium/well | no | no | MSS | no |  |  |
| P212 | 62 | Male | Left | II | Adeno | medium/well | no | no | MSS | yes |  |  |
| P213 | 62 | Female | Left | II | Adeno | poor | no | yes | MSS | no |  |  |
| P214 | 53 | Male | Left | III | Adeno | medium/well | no | no | MSS | yes |  |  |
| P215 | 45 | Male | Right | II | Mucinous | poor | no | no | MSI_H | no |  |  |
| P216 | 42 | Female | Left | III | Adeno | medium/well | no | no | MSS | yes |  |  |
| P217 | 64 | Male | Left | III | Adeno | medium/well | no | no | MSS | yes |  |  |
| P218 | 41 | Male | Left | III | Adeno | medium/well | no | no | MSI_H | yes |  |  |
| P219 | 45 | Female | Left | III | Adeno | medium/well | no | no | MSS | yes |  |  |
| P220 | 42 | Male | Left | III | Adeno | poor | no | no | MSS | yes |  |  |
| P221 | 65 | Male | Left | III | Adeno | medium/well | no | no | MSS | yes |  |  |
| P222 | 34 | Male | Right | II | Adeno | poor | no | no | MSS | yes |  |  |
| P223 | 41 | Male | Left | III | Adeno | medium/well | no | yes | MSS | yes |  |  |
| P224 | 60 | Male | Left | III | Adeno | medium/well | no | no | MSS | yes | 11.24 | Palliative |
| P225 | 68 | Female | Left | III | Adeno | medium/well | no | no | MSS | yes |  |  |
| P226 | 56 | Male | Right | III | Mucinous | poor | no | no | MSI_H | yes |  |  |
| P227 | 63 | Female | Left | III | Adeno | medium/well | no | yes | MSS | yes |  |  |
| P228 | 65 | Female | Left | III | Adeno | medium/well | no | yes | MSS | yes | 7.66 | Palliative |
| P229 | 63 | Female | Left | III | Adeno | medium/well | no | yes | MSS | no |  |  |
| P230 | 50 | Male | Left | III | Adeno | medium/well | no | yes | MSS | yes |  |  |
| P231 | 34 | Female | Right | III | Adeno | medium/well | no | yes | MSS | yes |  |  |
| P232 | 63 | Male | Right | III | Mucinous | poor | no | yes | MSS | yes |  |  |
| P233 | 51 | Female | Right | III | Adeno | medium/well | no | yes | MSI_H | yes |  |  |
| P234 | 69 | Male | Left | III | Adeno | medium/well | no | no | MSS | no |  |  |
| P235 | 51 | Female | Right | III | Mucinous | poor | no | no | MSS | yes |  |  |
| P236 | 69 | Female | Right | III | Adeno | medium/well | no | no | MSS | yes |  |  |
| P237 | 62 | Female | Left | III | Adeno | medium/well | no | no | MSS | yes |  |  |
| P238 | 47 | Female | Right | III | Adeno | medium/well | no | no | MSS | yes |  |  |
| P239 | 61 | Male | Left | III | Adeno | medium/well | no | no | MSS | yes |  |  |
| P240 | 55 | Female | Right | III | Adeno | poor | yes | yes | MSS | yes | 7.00 | Surgery |

a. PTL, primary tumor location. Right, right-sided; Left, left-sided.

b. Hist_type, histological type. Adeno, adenocarcinoma; Mucinous, mucinous or signet-ring carcinoma.

c. Hist_grade, histological grade.

d. LV_invasion, lymphovascular invasion.

e. N_invasion, nerve invasion.

f. ACT, adjuvant chemotherapy.

g. R_time, time from surgery to first documented recurrence.

h. R_treatment, treatment type after disease recurrence.
