# Supplementary material for: Postoperative circulating tumor DNA as markers of recurrence risk in stages II to III colorectal cancer
Source: J Hematol Oncol. 2021 May 17;14:80. doi: 10.1186/s13045-021-01089-z (PMC8130394; doi:10.1186/s13045-021-01089-z)
Supplement: Supplementary file 2 — Additional file 2: Table S2. Gene list of the Geneseeq Prime™ 425-gene panel. [file 13045_2021_1089_MOESM2_ESM.docx]

| **Table S2. Gene list of the Geneseeq Prime™ 425-gene panel.** | | | | | | | | | |
| --- | --- | --- | --- | --- | --- | --- | --- | --- | --- |
| ABCB1(MDR1) | BMPR1A | CUX1 | ETV6 | GSTP1 | LYN | NKX2-1 | PPARD | SDC4 | THADA |
| ABCB4 | BRAF | CXCR4 | EWSR1 | GSTT1 | LZTR1 | NKX2-4 | PPP2R1A | SDHA | TMEM127 |
| ABCC2(MRP2) | BRCA1 | CYLD | EXT1 | HDAC2 | MAP2K1(MEK1) | NOTCH1 | PRDM1 | SDHB | TMPRSS2 |
| ADH1A | BRCA2 | CYP19A1 | EXT2 | HDAC9 | MAP2K2(MEK2) | NOTCH2 | PRF1 | SDHC | TNFAIP3 |
| ADH1B | BRD4 | CYP2A13 | EZH2 | HGF | MAP2K4 | NOTCH3 | PRKACA | SDHD | TNFRSF11A |
| ADH1C | BRIP1 | CYP2A6 | FANCA | HLA-A | MAP3K1 | NPM1 | PRKACG | SEPT9  (SEPTIN9) | TNFRSF14 |
| AIP | BTG2 | CYP2A7 | FANCC | HNF1A | MAP3K4 | NQO1 | PRKAR1A | SETBP1 | TNFRSF19 |
| AKT1 | BTK | CYP2B6*6 | FANCD2 | HNF1B | MAP4K3 | NRAS | PRKCI | SETD2 | TNFSF11 |
| AKT2 | BUB1B | CYP2C19*2 | FANCE | HRAS | MAX | NRG1 | PRKDC | SF3B1 | TOP1 |
| AKT3 | c11orf30(EMSY) | CYP2C9*3 | FANCF | HSD3B1 | MCL1 | NSD1 | PRSS1 | SGK1 | TOP2A |
| ALDH2 | CASP8 | CYP2D6 | FANCG | IDH1 | MDM2 | NTRK1 | PRSS3 | SLC34A2 | TP53 |
| ALK | CBL | CYP3A4*4 | FANCI | IDH2 | MDM4 | NTRK2 | PTCH1 | SLC3A2 | TP63 |
| AMER1 | CBLB | CYP3A5 | FANCL | IFNG | MECOM | NTRK3 | PTEN | SLC7A8 | TPMT |
| APC | CCND1 | DAXX | FANCM | IFNGR1 | MED12 | PAK3 | PTK2 | SMAD2 | TSC1 |
| AR | CCNE1 | DDR2 | FAT1 | IGF1R | MEF2B | PALB2 | PTPN11 | SMAD3 | TSC2 |
| ARAF | CD274(PD-L1) | DENND1A | FBXW7 | IGF2 | MEN1 | PALLD | PTPN13 | SMAD4 | TSHR |
| ARID1A | CD74 | DHFR | FGF19 | IKBKE | MET | PARK2(PRKN) | PTPRD | SMAD7 | TTF1 |
| ARID1B | CDA | DICER1 | FGFR1 | IKZF1 | MGMT | PARP1 | QKI | SMARCA4 | TUBB3 |
| ARID2 | CDC73 | DLL3 | FGFR2 | IL7R | MITF | PARP2 | RAC1 | SMARCB1 | TUBB4A |
| ARID5B | CDH1 | DNMT3A | FGFR3 | INPP4B | MLH1 | PAX5 | RAC3 | SMO | TUBB4B |
| ASCL4 | CDK10 | DPYD | FGFR4 | IRF2 | MLH3 | PBRM1 | RAD50 | SOS1 | TUBB6 |
| ASXL1 | CDK12 | DUSP2 | FH | JAK1 | MLLT1 | PDCD1(PD1) | RAD51 | SOX1 | TYMS |
| ATF1 | CDK4 | EGFR | FLCN | JAK2 | MLLT3 | PDCD1LG2(PD-L2) | RAD51B | SOX14 | U2AF1 |
| ATIC | CDK6 | EML4 | FLT1  (VEGFR1) | JAK3 | MLLT4(AFDN) | PDE11A | RAD51C | SOX2 | UGT1A1 |
| ATM | CDK8 | EP300 | FLT3 | JARID2 | MPL | PDGFRA | RAD51D | SOX21 | VAMP2 |
| ATR | CDKN1A | EPAS1 | FLT4 | JUN | MRE11A  (MRE11) | PDGFRB | RAD54L | SPOP | VEGFA |
| ATRX | CDKN1B | EPCAM | FOXA1 | KDM5A | MSH2 | PDK1 | RAF1 | SPRY4 | VHL |
| AURKA | CDKN1C | EPHA2 | FOXP1 | KDM6A | MSH6 | PGR | RARA | SRC | WAS |
| AURKB | CDKN2A | EPHA3 | FRG1 | KDR  (VEGFR2) | MTHFR | PHOX2B | RARG | SRY | WISP3  (CCN6) |
| AXIN2 | CDKN2B | EPHA5 | GATA1 | KEAP1 | MTOR | PIK3C3 | RASGEF1A | STAG2 | WRN |
| AXL | CDKN2C | EPHB2 | GATA2 | KIF1B | MUTYH | PIK3CA | RB1 | STAT3 | WT1 |
| B2M | CEBPA | ERBB2  (HER2) | GATA3 | KIF5B | MYC | PIK3R1 | RECQL4 | STK11 | XPA |
| BAD | CEP57 | ERBB2IP  (ERBIN) | GATA4 | KIT | MYCL | PIK3R2 | RELN | STMN1 | XPC |
| BAI3  (ADGRB3) | CHD4 | ERBB3 | GATA6 | KITLG | MYCN | PKHD1 | RET | STT3A | XRCC1 |
| BAK1 | CHEK1 | ERBB4 | GNA11 | KLLN | MYD88 | PLAG1 | RHOA | SUFU | YAP1 |
| BAP1 | CHEK2 | ERCC1 | GNAQ | KMT2A(MLL) | MYH9 | PLK1 | RICTOR | TAP1 | ZNF2 |
| BARD1 | CREBBP | ERCC2 | GNAS | KMT2B | NAT1 | PMS1 | RNF43 | TAP2 | ZNF217 |
| BAX | CRKL | ERCC3 | GRIN2A | KMT2C | NBN | PMS2 | ROS1 | TEK | ZNF703 |
| BCL2 | CSF1R | ERCC4 | GRM3 | KMT2D  (MLL2) | NCOR1 | POLD1 | RPTOR | TEKT4 |  |
| BCL2L11(BIM) | CTCF | ERCC5 | GRM8 | KRAS | NF1 | POLD3 | RRM1 | TERC |  |
| BCR | CTLA4 | ESR1 | GSTM1 | LHCGR | NF2 | POLE | RUNX1 | TERT |  |
| BIRC3 | CTNNB1 | ETV1 | GSTM4 | LMO1 | NFE2L2 | POLH | RUNX1T1 | TET2 |  |
| BLM | CUL3 | ETV4 | GSTM5 | LRP1B | NFKBIA | POT1 | SBDS | TGFBR2 |  |
