# Supplementary material for: Postoperative circulating tumor DNA as markers of recurrence risk in stages II to III colorectal cancer
Source: J Hematol Oncol. 2021 May 17;14:80. doi: 10.1186/s13045-021-01089-z (PMC8130394; doi:10.1186/s13045-021-01089-z)
Supplement: Supplementary file 3 — Additional file 3: Methods. [file 13045_2021_1089_MOESM3_ESM.docx]

Supplementary Methods

**Additional information on study design and participants**

Patients clinically diagnosed as stage II to III CRC and willing to provide informed consent were recruited for study entry, and further confirmed for eligibility according to the following criteria, (i) patients who have previous malignancy within the last five years were excluded; (ii) patients presented with multiple primary tumors at diagnosis were excluded; (iii) patients who received treatment (chemotherapy or radiotherapy) before surgery were excluded; (iv) patients who withdrew informed consent at surgery were not included; (v) patients restaged as stage I or stage IV after surgery were excluded. Patients lost to follow-up immediately after surgery and patients with no somatic variants detected in the primary tumor tissue were further excluded in the final analysis. All tumor tissue and plasma samples were sequenced at a CLIA-certified and CAP-accredited clinical testing center of the Nanjing Geneseeq Technology Inc.. Serum CEA was tested by the local participating cancer centers, with a normal reference range of 0.00 to 5.00 ng/ml.

**Circulating tumor DNA analysis**

**Sample collection and DNA extractions.** Tumor tissue was collected at surgery for all patients. Genomic DNA from primary tumor tissues and peripheral blood leukocytes was extracted using QIAamp DNA FFPE Tissue Kit and DNeasy Blood & Tissue Kit (Qiagen), respectively. At each sampling point, 8 to 10 mL of peripheral blood was drawn into Cell-Free DNA BCT^®^ tubes (Streck) or K2-EDTA tubes (BD Biosciences), the plasma fraction was prepared within 2 hours of blood collection and shipped to the central testing laboratory within 72 hours. cfDNA was extracted using the QIAamp Circulating Nucleic Acid Kit (Qiagen). Quantification of cfDNA was performed using Qubit 3.0 Fluorometer with the dsDNA HS Assay Kit (Life Technologies).

**Library construction and targeted sequencing.** Sequencing libraries were constructed using the KAPA Hyper Library Prep Kit (KAPA Biosystems). Hybridization capture-based targeted enrichment was performed using Geneseeq Prime™ 425-gene panel as we previously described[[1](#_ENREF_1)], and the gene list was presented in Additional file 2: **Table S2,**. Libraries were quantified by qPCR using KAPA Library Quantification kit (KAPA Biosystems). Library fragment size was determined by Bioanalyzer 2100 (Agilent Technologies). The target-enriched library was then sequenced on the HiSeq4000 NGS platforms (Illumina). The mean sequencing depth was 276x for the peripheral blood leukocytes, 1277x for primary tumor tissues, and 4693x for plasma samples.

**Sequencing data processing and variant calling.** Fastp (v0.20.0)[[2](#_ENREF_2)] was used for quality control and adapter removal of the FastQ files. Leading/trailing low quality (quality score below 30) or N bases were removed. Sequencing reads were mapped to the hg19 reference genome using bwa-mem (v0.7.17-r1188)[[3](#_ENREF_3)]. PCR duplicates were marked using sambamba (v0.8.0)[[4](#_ENREF_4)]. Single nucleotide variants (SNVs) and small insertions/deletions (INDELs) were identified by VarScan2[[5](#_ENREF_5)] with tumor and matched normal DNA. Further filtering criteria were applied to the variants identified in the primary tumors, including (i) not present in the internal database of normal using peripheral blood leukocyte samples from ~500 healthy donors; (ii) present in <1% of the population in the 1000 Genomes Project (1000G)[[6](#_ENREF_6)], the Exome Aggregation Consortium (ExAC)[[7](#_ENREF_7)], and the Genome Aggregation Database (gnomAD)[[8](#_ENREF_8)]; (iii) variant allele frequency (VAF) ≥ 0.5%, supporting reads ≥ 3, depth ≥ 30x for recurrent variants (≥20 mentions in COSMIC v92[[9](#_ENREF_9)]), and VAF ≥ 1%, supporting reads ≥ 6, depth ≥ 30x for non-recurrent variants. All qualified variants identified in the primary tumor of each patient were regarded as patient-specific somatic variants for further ctDNA tracking (tracking variants hereafter). As to plasma variant calling, a set of cfDNA samples from 33 healthy donors were pre-processed to estimate the background VAF distribution for background polishing as previously described[[10](#_ENREF_10)]. For each tracking variant, the read depth of mutant and reference alleles in the plasma were used to calculate the *P* value against the background VAF distribution, and variant with a corresponding FDR-adjusted *P* value < 0.01 was retained as a true variant; we further required a minimum supporting reads of 3, a minimum depth of 100x for recurrent variants and a minimum supporting reads of 6, a minimum depth of 100x for non-recurrent variants. In addition, for ctDNA variants not present in the corresponding primary tumor, they were rescued as true variants to account for tumor heterogeneity and clonal evolution if the following stringent criteria were met, (i) VAF ≥ 1%, supporting reads ≥ 6, depth ≥ 100x; (ii) not present in our previously published database of clonal hematopoiesis variants[[11](#_ENREF_11)]; (iii) VAF = 0% in the paired peripheral blood leukocyte sample. Finally, to account for various number of tracking variants of different patients and reduce false positive, a sample was declared as ctDNA-positive only if the number of true variants detected in the plasma was more than 5% of the number of total tracking variants in each patient.

**Nomogram construction**

Clinicopathological risk factors, recurrent-mutated genes in the primary tumor and ctDNA status at day 3-7 postoperatively were considered in the construction of nomogram for postoperative risk stratification. Univariate Cox regression analysis was performed to identify clinicopathological risk factors that were significantly associated with recurrence-free survival (RFS). To select recurrent-mutated genes in the primary tumor that significantly associated with RFS, we used the Least Absolute Shrinkage and Selection Operator (LASSO) method in the Cox regression model as we previously described[[12](#_ENREF_12)]. Genes with mutational frequency above 5% were subjected to the LASSO Cox regression model, and we required selected genes to appear over 125 times out of a total 500 repetitions[[13](#_ENREF_13)].

Supplementary Reference

1. Jin Y, Chen DL, Wang F, Yang CP, Chen XX, You JQ, et al. The predicting role of circulating tumor DNA landscape in gastric cancer patients treated with immune checkpoint inhibitors. Mol Cancer 2020;19(1):154.

2. Chen S, Zhou Y, Chen Y, Gu J. fastp: an ultra-fast all-in-one FASTQ preprocessor. Bioinformatics 2018;34(17):i884-i90.

3. Li H. *Aligning sequence reads, clone sequences and assembly contigs with BWA-MEM*. Preprint at https://arxiv.org/abs/1303.3997v2. (2013).

4. Tarasov A, Vilella AJ, Cuppen E, Nijman IJ, Prins P. Sambamba: fast processing of NGS alignment formats. Bioinformatics 2015;31(12):2032-4.

5. Koboldt DC, Zhang Q, Larson DE, Shen D, McLellan MD, Lin L, et al. VarScan 2: somatic mutation and copy number alteration discovery in cancer by exome sequencing. Genome Res 2012;22(3):568-76.

6. Auton A, Brooks LD, Durbin RM, Garrison EP, Kang HM, Korbel JO, et al. A global reference for human genetic variation. Nature 2015;526(7571):68-74.

7. Karczewski KJ, Weisburd B, Thomas B, Solomonson M, Ruderfer DM, Kavanagh D, et al. The ExAC browser: displaying reference data information from over 60 000 exomes. Nucleic Acids Res 2017;45(D1):D840-d45.

8. Karczewski KJ, Francioli LC, Tiao G, Cummings BB. The mutational constraint spectrum quantified from variation in 141,456 humans. Nature 2020;581(7809):434-43.

9. Tate JG, Bamford S, Jubb HC, Sondka Z, Beare DM, Bindal N, et al. COSMIC: the Catalogue Of Somatic Mutations In Cancer. Nucleic Acids Res 2019;47(D1):D941-d47.

10. Newman AM, Lovejoy AF, Klass DM, Kurtz DM, Chabon JJ, Scherer F, et al. Integrated digital error suppression for improved detection of circulating tumor DNA. Nat Biotechnol 2016;34(5):547-55.

11. Li Z, Huang W, Yin JC. Comprehensive next-generation profiling of clonal hematopoiesis in cancer patients using paired tumor-blood sequencing for guiding personalized therapies. Clin Transl Med 2020;10(7):e222.

12. Ju HQ, Zhao Q, Wang F, Lan P, Wang Z, Zuo ZX, et al. A circRNA signature predicts postoperative recurrence in stage II/III colon cancer. EMBO Mol Med 2019;11(10):e10168.

13. Xu RH, Wei W, Krawczyk M, Wang W, Luo H, Flagg K, et al. Circulating tumour DNA methylation markers for diagnosis and prognosis of hepatocellular carcinoma. Nat Mater 2017;16(11):1155-61.
