# Supplementary material for: Postoperative circulating tumor DNA as markers of recurrence risk in stages II to III colorectal cancer
Source: J Hematol Oncol. 2021 May 17;14:80. doi: 10.1186/s13045-021-01089-z (PMC8130394; doi:10.1186/s13045-021-01089-z)
Supplement: Supplementary file 4 — Additional file 4: Table S3. Baseline characteristics of 240 evaluable patients. [file 13045_2021_1089_MOESM4_ESM.docx]

| **Characteristics** | **No. (%)** |
| --- | --- |
| **No. of patients** | 240 |
| **Median follow-up (months), 95%CI** | 27.4 (26.2, 28.5) |
| **Median age, years (range)** | 60 (19, 84) |
| **Sex** |  |
| Female | 104 (43.3) |
| Male | 136 (56.7) |
| **Primary tumor location** |  |
| Left-sided | 153 (63.7) |
| Right-sided | 87 (36.3) |
| **Pathological stage** |  |
| II | 112 (46.7) |
| III | 128 (53.3) |
| **Histological type** |  |
| Adenocarcinoma | 212 (88.3) |
| Mucinous/signet-ring carcinoma | 28 (11.7) |
| **Histological grade** |  |
| Moderate/well | 180 (75.0) |
| Poor | 60 (25.0) |
| **Adjuvant chemotherapy by stage** |  |
| II | 53 (47.3) |
| III | 121 (94.5) |
| **Relapse by stage** |  |
| II | 10 (8.9) |
| III | 22 (17.2) |
| **MSI status** |  |
| MSI-H | 23 (9.6) |
| MSI-L/MSS | 217 (90.4) |

**Table S3. Baseline characteristics of 240 evaluable patients.**
