# Supplementary material for: Postoperative circulating tumor DNA as markers of recurrence risk in stages II to III colorectal cancer
Source: J Hematol Oncol. 2021 May 17;14:80. doi: 10.1186/s13045-021-01089-z (PMC8130394; doi:10.1186/s13045-021-01089-z)

Figure S1. Heatmap demonstrating the mutational landscape of the primary tumors from 240 evaluable patients, with the upper third depicting the mutation counts of each patient, the middle third presenting the top 20 recurrent-mutated genes in this cohort, and the lower third showing the corresponding clinicopathological features.

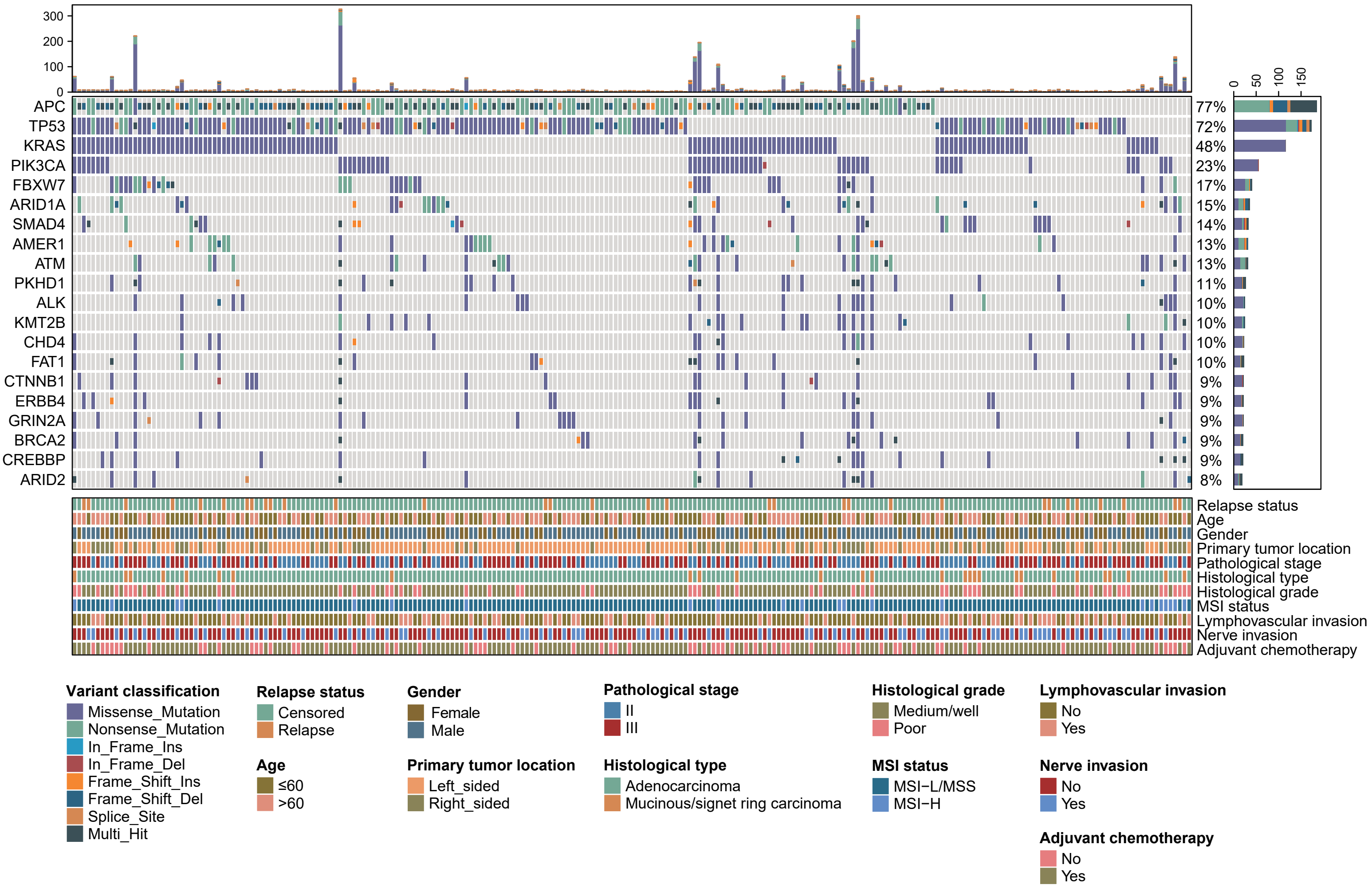

Supplement: Supplementary file 5 — Additional file 5: Figure S1. Heatmap demonstrating the mutational landscape of the primary tumors from 240 evaluable patients, with the upper third depicting the mutation counts of each patient, the middle third presenting the top 20 recurrent-mutated genes in this cohort, and the lower third showing the corresponding clinicopathological features. [file 13045_2021_1089_MOESM5_ESM.pdf]
