# Supplementary material for: Postoperative circulating tumor DNA as markers of recurrence risk in stages II to III colorectal cancer
Source: J Hematol Oncol. 2021 May 17;14:80. doi: 10.1186/s13045-021-01089-z (PMC8130394; doi:10.1186/s13045-021-01089-z)

Figure S2. Preoperative examination of CEA and ctDNA in 240 evaluable patients stratified by pathological stage. (Nine patients with no CEA information available pre-operation)

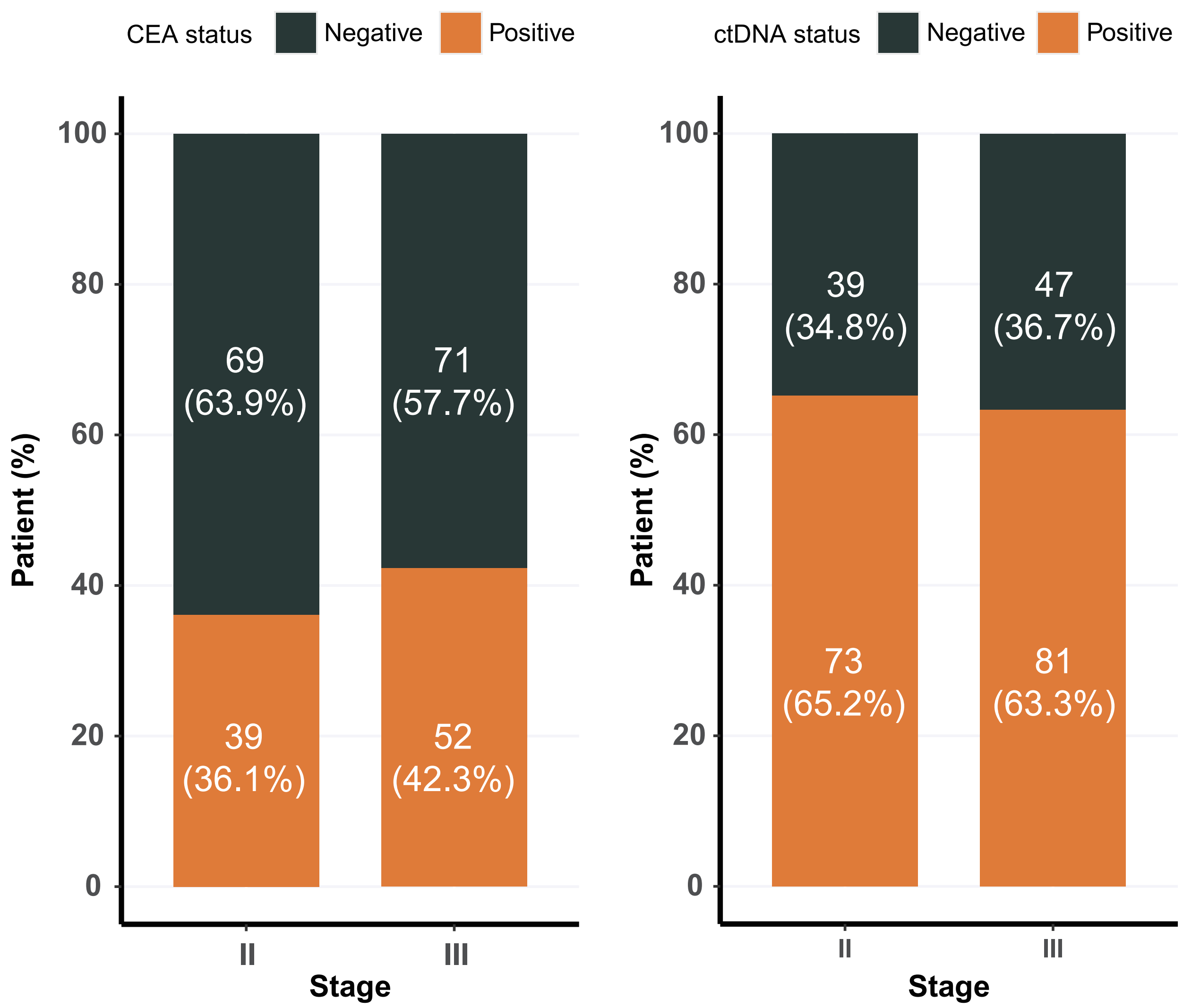

Supplement: Supplementary file 8 — Additional file 8: Figure S2. Preoperative examination of CEA and ctDNA in 240 evaluable patients stratified by pathological stage. (Nine patients with no CEA information available pre-operation). [file 13045_2021_1089_MOESM8_ESM.pdf]
