# Supplementary material for: Postoperative circulating tumor DNA as markers of recurrence risk in stages II to III colorectal cancer
Source: J Hematol Oncol. 2021 May 17;14:80. doi: 10.1186/s13045-021-01089-z (PMC8130394; doi:10.1186/s13045-021-01089-z)
Supplement: Supplementary file 10 — Additional file 10: Figure S3. Kaplan–Meier estimates of recurrence-free survival (RFS) according to preoperative CEA and ctDNA status. [file 13045_2021_1089_MOESM10_ESM.pdf]

Figure S3. Kaplan-Meier estimates of recurrence-free survival (RFS) according to preoperative CEA and ctDNA status.

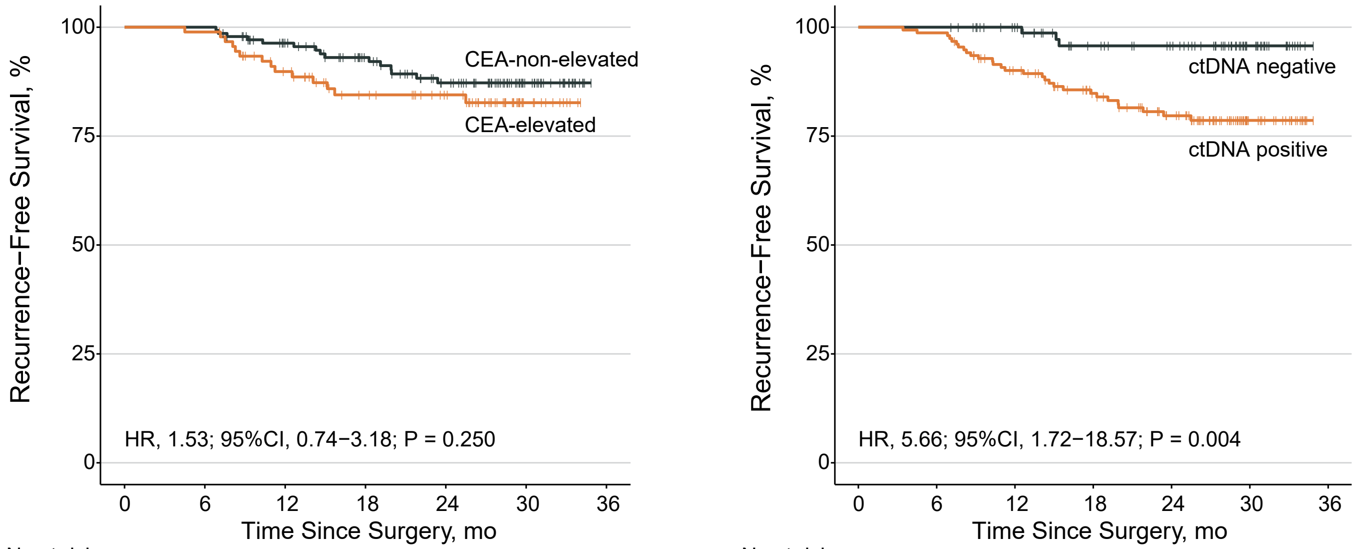

No.at risk	0	6	12	18	24	30	36
CEA-non-elevated	140	140	121	101	81	30	0
CEA-elevated	91	90	75	58	49	14	0

No.at risk	0	6	12	18	24	30	36
ctDNA negative	86	86	76	61	54	24	0
ctDNA positive	154	152	128	104	82	21	0
