# Supplementary material for: Postoperative circulating tumor DNA as markers of recurrence risk in stages II to III colorectal cancer
Source: J Hematol Oncol. 2021 May 17;14:80. doi: 10.1186/s13045-021-01089-z (PMC8130394; doi:10.1186/s13045-021-01089-z)
Supplement: Supplementary file 11 — Additional file 11: Table S4. Patients’ clinical features stratified by preoperative ctDNA status. [file 13045_2021_1089_MOESM11_ESM.docx]

**Table S4. Patients’ clinical features stratified by preoperative ctDNA status.**

| **Characteristics** | **ctDNA-negative** | **ctDNA-positive** | ***P*** |
| --- | --- | --- | --- |
| **No. of patients** | 86 (35.8%) | 154 (64.2%) | - |
| **Age (median[IQR])** | 62.0 [53.3, 66.8] | 58.5 [49.3, 65.0] | 0.063 |
| **Sex** |  |  | 0.892 |
| Female | 38 (44.2) | 66 (42.9) |  |
| Male | 48 (55.8) | 88 (57.1) |  |
| **Primary tumor location** |  |  | 1 |
| Left-sided | 55 (64.0) | 98 (63.6) |  |
| Right-sided | 31 (36.0) | 56 (36.4) |  |
| **T stage** |  |  | 0.221 |
| 1 | 2 (2.3) | 1 (0.6) |  |
| 2 | 5 (5.8) | 3 (1.9) |  |
| 3 | 66 (76.7) | 122 (79.2) |  |
| 4 | 13 (15.1) | 28 (18.2) |  |
| **N stage** |  |  | 0.788 |
| N (-) | 39 (45.3) | 73 (47.4) |  |
| N (+) | 47 (54.7) | 81 (52.6) |  |
| **Histological type** |  |  | 0.834 |
| Adenocarcinoma | 77 (89.5) | 135 (87.7) |  |
| Mucinous/signet-ring carcinoma | 9 (10.5) | 19 (12.3) |  |
| **Histological grade** |  |  | 0.756 |
| Moderate/well | 66 (76.7) | 114 (74.0) |  |
| Poor | 20 (23.3) | 40 (26.0) |  |
| **Lymphovascular invasion** |  |  | 0.768 |
| No | 62 (72.1) | 107 (69.5) |  |
| Yes | 24 (27.9) | 47 (30.5) |  |
| **Nerve invasion** |  |  | 1 |
| No | 55 (64.0) | 97 (63.0) |  |
| Yes | 31 (36.0) | 57 (37.0) |  |
| **MSI status** |  |  | 0.366 |
| MSI-H | 6 (7.0) | 17 (11.0) |  |
| MSI-L/MSS | 80 (93.0) | 137 (89.0) |  |
