# Supplementary material for: Postoperative circulating tumor DNA as markers of recurrence risk in stages II to III colorectal cancer
Source: J Hematol Oncol. 2021 May 17;14:80. doi: 10.1186/s13045-021-01089-z (PMC8130394; doi:10.1186/s13045-021-01089-z)

Figure S4. The clinical courses together with ctDNA statuses of 8 out of 20 ctDNA-positive (at day 3-7 post-operation) patients without documented recurrence.

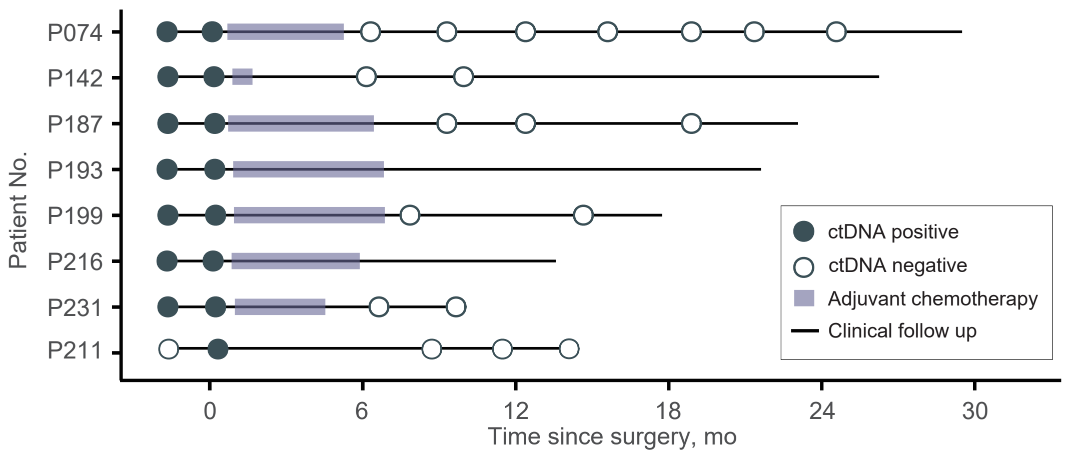

Supplement: Supplementary file 12 — Additional file 12: Figure S4. The clinical courses together with ctDNA statuses of 8 out of 20 ctDNA-positive (at day 3–7 postoperation) patients without documented recurrence. [file 13045_2021_1089_MOESM12_ESM.pdf]
