# Supplementary material for: Postoperative circulating tumor DNA as markers of recurrence risk in stages II to III colorectal cancer
Source: J Hematol Oncol. 2021 May 17;14:80. doi: 10.1186/s13045-021-01089-z (PMC8130394; doi:10.1186/s13045-021-01089-z)

Figure S5. Subgroup analysis of the association between ctDNA status and recurrence-free survival in patients receiving adjuvant chemotherapy or not.

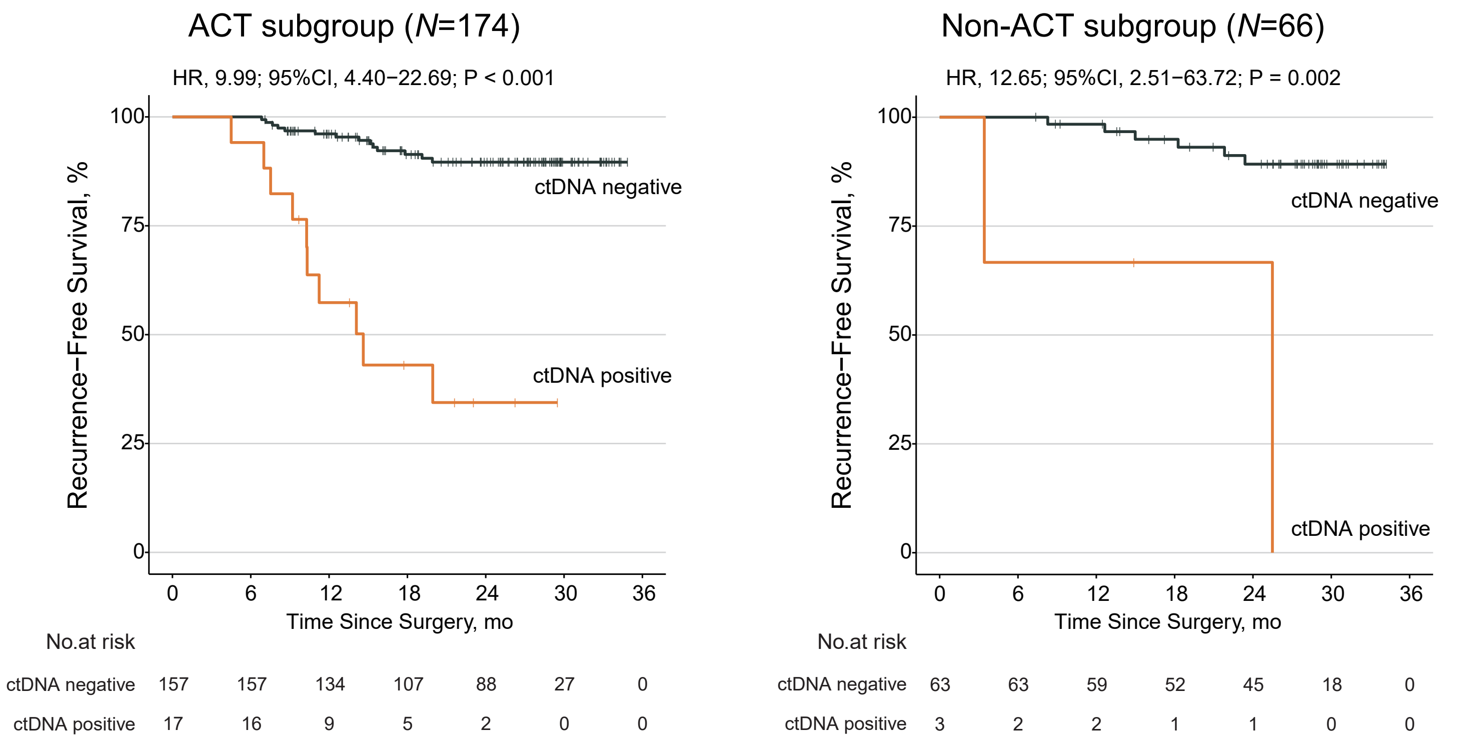

Supplement: Supplementary file 13 — Additional file 13: Figure S5. Subgroup analysis of the association between ctDNA status and recurrence-free survival in patients receiving adjuvant chemotherapy or not. [file 13045_2021_1089_MOESM13_ESM.pdf]
