# Supplementary material for: Postoperative circulating tumor DNA as markers of recurrence risk in stages II to III colorectal cancer
Source: J Hematol Oncol. 2021 May 17;14:80. doi: 10.1186/s13045-021-01089-z (PMC8130394; doi:10.1186/s13045-021-01089-z)
Supplement: Supplementary file 14 — Additional file 14: Table S5. Univariate and multivariable Cox analysis of recurrence-free survival by clinicopathological variables and ctDNA status at first sampling point after adjuvant chemotherapy. [file 13045_2021_1089_MOESM14_ESM.docx]

**Table S5. Univariate and multivariable Cox analysis of recurrence-free survival by clinicopathological variables and ctDNA status at first sampling point after adjuvant chemotherapy (ACT).**

| **Variable** | **Univariate analysis** | | **Multivariable analysis** | |
| --- | --- | --- | --- | --- |
|  | **HR (95% CI)** | ***P**** | **HR (95% CI)** | ***P**** |
| **Age, years** |  |  |  |  |
| ≤60 versus >60 | 2.12 (0.83-5.42) | 0.117 |  |  |
| **Sex** |  |  |  |  |
| Male versus Female | 0.56 (0.24-1.30) | 0.176 |  |  |
| **Primary tumor location** |  |  |  |  |
| Right-sided versus Left-sided | 1.94 (0.83-4.54) | 0.127 |  |  |
| **Pathological stage** |  |  |  |  |
| III versus II | 11.80 (1.59-87.75) | **0.016** | 25.25 (2.93-217.39) | **0.003** |
| **Lymphovascular invasion** |  |  |  |  |
| Yes versus No | 3.73 (1.46-9.53) | **0.006** | 1.97 (0.68-5.71) | 0.214 |
| **Nerve invasion** |  |  |  |  |
| Yes versus No | 2.29 (0.96-5.45) | 0.062 |  |  |
| **Histological type** |  |  |  |  |
| Mucinous versus Adenocarcinoma | 1.21 (0.28-5.19) | 0.796 |  |  |
| **Histological grade** |  |  |  |  |
| Poor versus Medium/well | 0.98 (0.36-2.67) | 0.975 |  |  |
| **MSI status** |  |  |  |  |
| MSI-L/ MSS versus MSI-H | - | - |  |  |
| **CEA status** |  |  |  |  |
| Elevated versus Non-elevated | 3.54 (1.12-11.12) | **0.031** | 2.22 (0.64-7.68) | 0.206 |
| **Preoperative ctDNA status** |  |  |  |  |
| Positive versus Negative | 3.61 (1.07-12.20) | **0.039** | 1.34 (0.35-2.08) | 0.670 |
| **ctDNA status at first time point after ACT** |  |  |  |  |
| Positive versus Negative | 12.76 (5.39-30.19) | **<0.001** | 20.79 (6.66-64.87) | **<0.001** |

**P* value in bold denotes statistically significant.
