# Supplementary material for: Postoperative circulating tumor DNA as markers of recurrence risk in stages II to III colorectal cancer
Source: J Hematol Oncol. 2021 May 17;14:80. doi: 10.1186/s13045-021-01089-z (PMC8130394; doi:10.1186/s13045-021-01089-z)
Supplement: Supplementary file 15 — Additional file 15: Table S6. Univariate and multivariable Cox analysis of recurrence-free survival by clinicopathological variables and ctDNA status in surveillance samples. [file 13045_2021_1089_MOESM15_ESM.docx]

**Table S6. Univariate and multivariable Cox analysis of recurrence-free survival by clinicopathological variables and ctDNA status in surveillance samples.**

| **Variable** | **Univariate analysis** | | **Multivariable analysis** | |
| --- | --- | --- | --- | --- |
|  | **HR (95% CI)** | ***P**** | **HR (95% CI)** | ***P**** |
| **Age, years** |  |  |  |  |
| ≤60 versus >60 | 1.63 (0.69-3.85) | 0.263 |  |  |
| **Sex** |  |  |  |  |
| Male versus Female | 0.70 (0.31-1.61) | 0.404 |  |  |
| **Primary tumor location** |  |  |  |  |
| Right-sided versus Left-sided | 1.76 (0.77-3.98) | 0.177 |  |  |
| **Pathological stage** |  |  |  |  |
| III versus II | 5.73 (1.95-16.85) | **0.002** | 5.59 (1.59-19.62) | **0.007** |
| **Lymphovascular invasion** |  |  |  |  |
| Yes versus No | 2.88 (1.25-6.67) | **0.013** | 0.86 (0.32-2.33) | 0.729 |
| **Nerve invasion** |  |  |  |  |
| Yes versus No | 2.13 (0.93-4.86) | 0.072 |  |  |
| **Histological type** |  |  |  |  |
| Mucinous versus Adenocarcinoma | 0.97 (0.23-4.16) | 0.972 |  |  |
| **Histological grade** |  |  |  |  |
| Poor versus Medium/well | 1.01 (0.40-2.57) | 0.976 |  |  |
| **MSI status** |  |  |  |  |
| MSI-L/ MSS versus MSI-H | 1.88 (0.25-13.96) | 0.536 |  |  |
| **CEA status** |  |  |  |  |
| Elevated versus Non-elevated | 9.79 (3.75-25.56) | **<0.001** | 4.89 (1.72-13.92) | **0.003** |
| **Preoperative ctDNA status** |  |  |  |  |
| Positive versus Negative | 3.72 (1.11-12.52) | **0.034** | 1.39 (0.38-5.12) | 0.618 |
| **Serial ctDNA status** |  |  |  |  |
| Positive versus Negative | 32.02 (10.79-95.08) | **<0.001** | 23.00 (6.30-69.97) | **<0.001** |

**P* value in bold denotes statistically significant.
