# Supplementary material for: Postoperative circulating tumor DNA as markers of recurrence risk in stages II to III colorectal cancer
Source: J Hematol Oncol. 2021 May 17;14:80. doi: 10.1186/s13045-021-01089-z (PMC8130394; doi:10.1186/s13045-021-01089-z)

Figure S6. The clinical courses together with ctDNA statuses of 102 censored patients included in serial post-definitive-treatment ctDNA analysis (postoperative ctDNA sampling points after 27 months, which were all negative, were omitted in the plot).

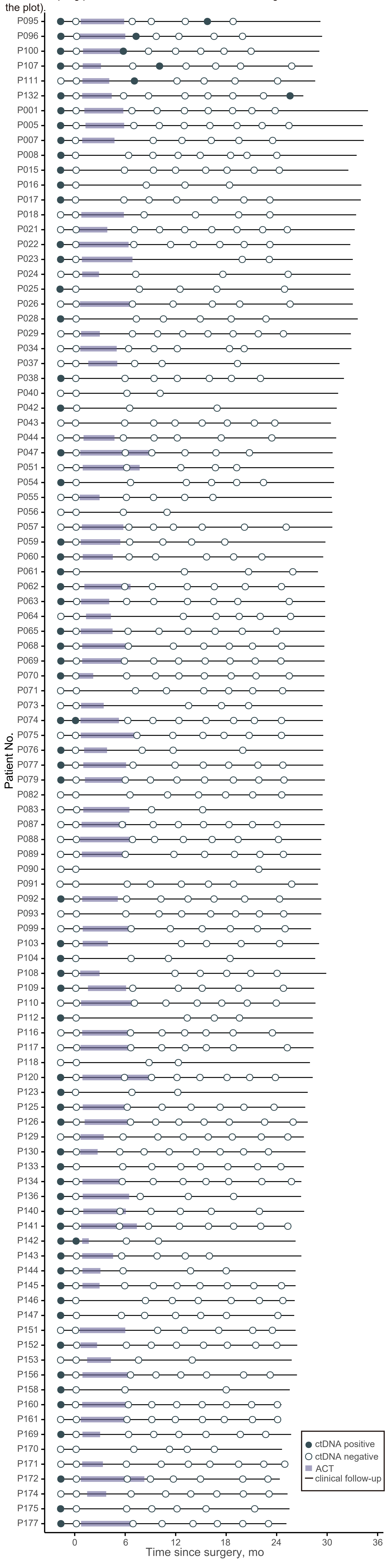

Supplement: Supplementary file 16 — Additional file 16: Figure S6. The clinical courses together with ctDNA statuses of 102 censored patients included in serial post-definitive treatment ctDNA analysis. [file 13045_2021_1089_MOESM16_ESM.pdf]
