# Supplementary material for: Postoperative circulating tumor DNA as markers of recurrence risk in stages II to III colorectal cancer
Source: J Hematol Oncol. 2021 May 17;14:80. doi: 10.1186/s13045-021-01089-z (PMC8130394; doi:10.1186/s13045-021-01089-z)

Figure S7. Comparison of time from surgery to disease recurrence by CEA and computed tomography (CT) scanning, dashed lines indicate mean time of recurrence based on CT (13.46 months) and CEA (12.08 months). (*N*=14, Student's t-test)

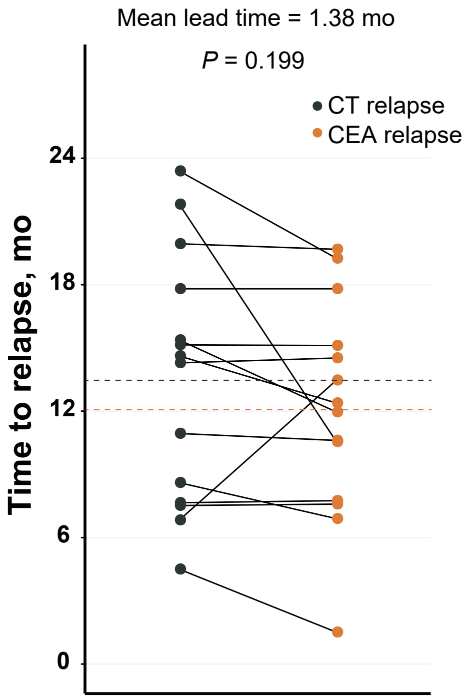

Supplement: Supplementary file 17 — Additional file 17: Figure S7. Comparison of time from surgery to disease recurrence by CEA and computed tomography (CT) scanning, dashed lines indicate mean time of recurrence based on CT (13.46 months) and CEA (12.08 months). (N=14, Student’s t-test) [file 13045_2021_1089_MOESM17_ESM.pdf]
