# Supplementary material for: Postoperative circulating tumor DNA as markers of recurrence risk in stages II to III colorectal cancer
Source: J Hematol Oncol. 2021 May 17;14:80. doi: 10.1186/s13045-021-01089-z (PMC8130394; doi:10.1186/s13045-021-01089-z)

Figure S8. In 9 patients with ≥2 ctDNA-positive plasma samples after definite treatment, the mean variant allele frequency (VAF) of their ctDNA in the plasma were demonstrated from first ctDNA detection to radiological confirmation of relapse (early time points before and during adjuvant chemotherapy were omitted). Each colored curve indicates data of one patient.

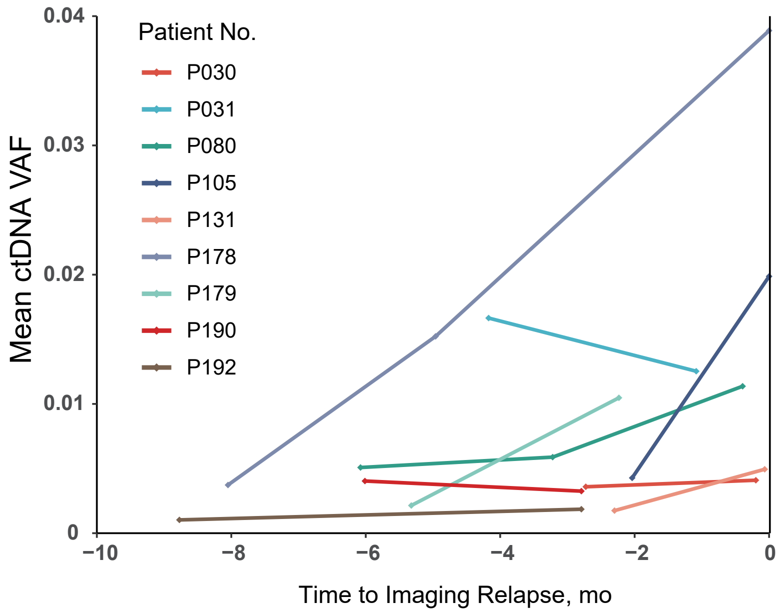

Supplement: Supplementary file 18 — Additional file 18: Figure S8. In 9 patients with ≥2 ctDNA-positive plasma samples after definite treatment, the mean variant allele frequency (VAF) of their ctDNA in the plasma was demonstrated from first ctDNA detection to radiological confirmation of relapse (early time points before and during adjuvant chemotherapy were omitted). Each colored curve indicates data of one patient. [file 13045_2021_1089_MOESM18_ESM.pdf]
